# Protein Subcellular Relocalization of Duplicated Genes in *Arabidopsis*

**DOI:** 10.1093/gbe/evu191

**Published:** 2014-09-04

**Authors:** Shao-Lun Liu, An Qi Pan, Keith L. Adams

**Affiliations:** ^1^Department of Botany, University of British Columbia, Vancouver, British Columbia, Canada; ^2^Present address: Department of Life Science, Tunghai University, Taichung, Taiwan; ^3^Present address: Mintec Inc., Vancouver, BC, Canada

**Keywords:** gene duplication, whole genome duplication, subcellular localization

## Abstract

Gene duplications during eukaroytic evolution, by successive rounds of polyploidy and by smaller scale duplications, have provided an enormous reservoir of new genes for the evolution of new functions. Preservation of many duplicated genes can be ascribed to changes in sequences, expression patterns, and functions. Protein subcellular relocalization (protein targeting to a new location within the cell) is another way that duplicated genes can diverge. We studied subcellular relocalization of gene pairs duplicated during the evolution of the Brassicaceae including gene pairs from the alpha whole genome duplication that occurred at the base of the family. We analyzed experimental localization data from green fluorescent protein experiments for 128 duplicate pairs in *Arabidopsis thaliana*, revealing 19 pairs with subcellular relocalization. Many more of the duplicate pairs with relocalization than with the same localization showed an accelerated rate of amino acid sequence evolution in one duplicate, and one gene showed evidence for positive selection. We studied six duplicate gene pairs in more detail. We used gene family analysis with several pairs to infer which gene shows relocalization. We identified potential sequence mutations through comparative analysis that likely result in relocalization of two duplicated gene products. We show that four cases of relocalization have new expression patterns, compared with orthologs in outgroup species, including two with novel expression in pollen. This study provides insights into subcellular relocalization of evolutionarily recent gene duplicates and features of genes whose products have been relocalized.

## Introduction

Gene duplication has been an important genetic process for producing morphological and physiological innovations during eukaryotic evolution (reviewed in Flagel and Wendel 2009; Fawcett and Van de Peer 2010). Duplicated genes are formed by several molecular mechanisms, including whole genome (WG) duplication, segmental duplication of various sized regions of chromosomes, tandem duplication, and dispersed duplications generated by RNA or DNA-based mechanisms. Many WG duplication events have occurred during angiosperm evolution (e.g., Jiao et al. 2011; Schnable et al. 2012). About 2,500 pairs of duplicated genes were derived from the most recent WG duplication event in the *Arabidopsis* lineage, referred to as the alpha WG (α-WG) duplication event, that occurred at or near the base of the Brassicaceae family (Blanc et al. 2003; Barker et al. 2009). There are many examples of other types of duplicates, such as tandem duplicates, that are specific to the Brassicaceae family (Haberer et al. 2004; Audemard et al. 2012).

A considerable number of duplicated genes have been retained in most flowering plants. Genes that are not dosage balanced may show neofunctionalization or subfunctionalization. In subfunctionalization, ancestral functions and/or expression patterns are divided between the two duplicates (Force et al. 1999; Lynch and Force 2000). In contrast, neofunctionalization occurred when one copy acquired a new function or expression pattern and the other copy retained the ancestral expression pattern or function. In addition to neofunctionalization and subfunctionalization of expression patterns and functions, protein subcellular relocalization after gene duplication recently began to receive more attention (Byun-McKay and Geeta 2008; Marques et al. 2008; Byun-McKay et al. 2009). Subcellular relocalization has been suggested to be an important type of molecular mechanism contributing to the preservation and functional divergence of duplicated genes in eukaryotic genomes (Byun and Singh 2013). Similar to the concepts of neofunctionalization and subfunctionalization, the protein product of a duplicated gene can acquire a new subcellular localization (referred to as neolocalization) or show dividing of ancestral subcellular localizations if there were two or more (referred to as sublocalization). After neolocalization or sublocalization, expression patterns and functions could also diverge.

A few cases of protein subcellular relocalization after gene duplication have been shown in plants. For example, a pair of coproporphyrinogen III oxidases, *CPX1* and *CPX2*, in maize shows differential targeting ability to chloroplasts and mitochondria (Williams et al. 2006). A more ancient case of subcellular relocalization is a pair of nucleus-encoded organellar ribosomal protein genes (*Rps13*) in rosids where one is localized to the chloroplast and the other has been relocalized to the mitochondria (Adams et al. 2002; Mollier et al. 2002). In addition, genes within a family with different subcellular localization have been identified (e.g., Schultz and Coruzzi 1995; Devoto et al. 1999; Heilmann et al. 2004; Kawashima et al. 2005; Murcha et al. 2007; Dixon et al. 2009; Chong et al. 2010; Lan et al. 2010), but in most cases the duplication history and sequence evolution were not studied. A large-scale evolutionary study of subcellular localization of many duplicate gene pairs, using experimental data, has not been conducted in plants.

In this study, we used experimental green fluorescent protein (GFP) data to assemble a set of duplicated genes with subcellular localization data available for both copies. That allowed us to identify cases of subcellular relocalization after gene duplication during the evolution of the Brassicacaeae family and determine if genes whose products have been relocalized more often show accelerated and asymmetric amino acid sequence evolution compared with those pairs that do not show relocalization. In addition, we studied six cases in more detail to infer which gene in a duplicate pair has been relocalized, to further characterize sequence rate evolution in the relocalized genes, to determine whether the relocalized genes show changes in expression patterns, and to infer sequence changes that may have led to relocalization in some cases with sufficient available localization data.

## Materials and Methods

### Subcellular Localization Analysis

To examine the subcellular localization of *PRX36* (At3g50990) and *PRX72* (At5g55390), full length cDNA products were amplified by reverse transcription PCR (RT-PCR) using gene-specific primers that include the following underlined restriction enzyme site: At3g50990F-*Kpn*I (5′-CGGGGTACCATGAATACAAAAACGGTGAAG-3′), At3g50990R-*Bam*HI (5′-CGCGGATCCAACATCATGGTTAACCCTCC-3′), At5g66390F-*Kpn*I (5′-CGGGGTACCATGGCCAAGTCATTGAACATC-3′), and At5g66390R-*Bam*HI (5′-CGCGGATCCATAAGCATGGTTAACCCTCC-3′), with the RT-PCR conditions described in Liu and Adams (2008). All PCR products were cloned in frame into modified pCambia1300 vectors with the CaMV 35S promoter located 5′ upstream and GFP located immediately downstream. The inserted nucleotide sequence in the resultant plasmid was checked by DNA sequencing. The pCambia1300-At3g50990-GFP and pCambia1300-At5g66390-GFP then were transformed into *Arabidopsis thaliana* ecotype Columbia. *Agrobacterium*-mediated transformation was conducted using the floral dip method described in Clough and Bent (1998). A small piece of transgenic plant tissue was placed on the slide and immersed in a few drops of sterile Milli-Q water for visualizing the GFP fluorescence by using a confocal laser scanning microscope.

### Plant Materials, Nucleic Acid Extraction, and RT-PCR

Different organ types (e.g., roots, rosettes, shoots, leaves, flowers, or siliques/seeds) of *Gossypium hirsutum* cultivar TM1, *Carica papaya* cultivar Sun-Up, and *A. thaliana* ecotype Columbia were harvested and then stored in a −80 °C freezer for the subsequent nucleic acid extraction and RT-PCR assays. Nucleic acid extraction and RT-PCR followed the procedures described in Liu and Adams (2008). Gene-specific primers for RT-PCR are listed in the supplementary table S1, Supplementary Material online. Gene-specific primers for gene annotation of *CPK* in *C. papaya* are listed in the supplementary table S2, Supplementary Material online. The partial sequence *CPK* from *C. papaya* determined in this study was submitted to GenBank with the accession number KC692920.

### Identification of Brassicaceae-Specific Duplicated Genes

Three different types of duplicates in *A. thaliana* were included: α-WG duplicates, tandem duplicates, and “other duplicates” that arose from the other types of gene duplication events. The α-WG duplicates were obtained from the study of Blanc et al. (2003). Tandem duplicates were obtained from Haberer et al. (2004), in which only clusters of two genes were selected to simplify further analyses. The “other duplicates” were identified using the following analytical procedure. First, we obtained *A. thaliana* gene families from PLAZA 2.5 (Proost et al. 2009) and then a consensus gene family tree for each gene family was generated from 100 bootstrapped maximum-likelihood (ML) trees constructed using the software RAxML (Stamatakis 2006). Second, each monophyletic group in the 50% majority consensus tree that only consists of two members was selected. Finally, the pairs of α-WG and tandem duplicates were filtered out, resulting in the list of other duplicates. In addition, we further filtered out older duplicates that were potentially derived from the duplication events shared with other species outside the family Brassicaceae because we aimed to include the Brassicaceae-specific duplicates. As synonymous substitution values (d*S*) between duplicate genes can be used as proxy of the age of duplication (e.g., Blanc et al. 2003), we used the d*S* value to filter out pairs that were older than the average age of the alpha duplicates. A d*S* of one was used as the cut off because the mean d*S* value of all α-WG duplicates is approximately 1 (Blanc et al. 2003). The d*S* values were computed with an ML method using the software Codeml in PAML 4 (Yang 2007). The final list of duplicates used in this study is provided in supplementary table S1, Supplementary Material online.

### Identification of Gene Pairs with GFP Localization Data Available for Both Duplicates

We first retrieved the subcellular localization data of *Arabidopsis* genes from the SUB-cellular location database for *Arabidopsis* proteins (SUBA database) (Heazlewood et al. 2007). The SUBA database integrated the subcellular localization data in *A. thaliana* from previously published papers. The subcellular localization data from the GFP approach have been shown to be more accurate than other computational prediction-based approaches (Heazlewood et al. 2005). Thus, only the GFP data were used in our study. We also performed a literature search to find duplicate genes with GFP data that were published after the last update of SUBA. To minimize false positives, we then performed several filtration steps to exclude some pairs for subsequent analyses. Several filtration steps were applied to minimize the false positives or exclude uncertain cases (see supplementary fig. S1, Supplementary Material online, for details). Protein subcellular localization has been shown to change under different environmental conditions (e.g., Alinsug et al. 2009), vary by organ type (e.g, Székely et al. 2008), and vary in an age-dependent manner (e.g., Teng et al. 2012). Thus, we excluded gene pairs where the GFP data come from different studies using different experimental conditions.

### Identification of Orthologs in Outgroup Species

Prior to the asymmetric sequence rate analyses, we first identified the orthologs for 128 pairs of duplicates with localization data. To this end, reciprocal best hits of BLASTP (RBH-BLASTP) between *Arabidopsis* and other eudicots as outgroups were performed. The outgroup protein sequences of *C. papaya*, *Fragaria vesca*, *Glycine max*, *Lotus japonica*, *Malus domestica*, *Manihot esculenta*, *Medicago truncatula*, *Populus trichocarpa*, *Ricinus communis*, *Theobroma cacao*, and *Vitis vinifera* were downloaded from PLAZA 2.5 (Proost et al. 2009). Then, the results of RBH-BLASTP were filtered according to the following criteria: 1) The *e* value was less than or equal to 1 × 10^−^^5^; and 2) the alignment length for the amino acid of one protein must be at least 60% of the shorter protein. If no orthologs were identified for a duplicated gene pair through RBH-BLASTP, putative orthologs identified by one way best hit were used. To avoid the identification of false positives, we then applied the second filtration step to ensure the identification of orthologous sequences from outgroup species. The divergence time between two duplicated genes should be younger than those between duplicates and their preduplication orthologs. To this end, the pairwise d*S* values were calculated to examine whether an appropriate orthologous sequence was chosen. First, coding sequences were aligned by codons using the software MUSCLE (Edgar 2004). Second, the d*S* value of two duplicated genes (d*S*_1_), the duplicate gene 1 and the ortholog (d*S*_2_), and the duplicate gene 2 and the ortholog (d*S*_3_) were computed using the software Codeml in PAML 4 (Yang 2007). Third, only pairs with d*S*_2_ > d*S*_1_ and d*S*_3_ > d*S*_1_ were kept for the asymmetric sequence rate analysis based on our assumption for the relationship between the duplicated genes and their orthologs.

To retrieve the orthologs from outgroup species for each case study prior to asymmetric rate analysis, we first retrieved orthologs of duplicated genes from the PLAZA 2.5 orthologs list (Proost et al. 2009), then reconstructed an ML gene family phylogenetic tree using MEGA5 with assessment of the statistical node support by 500 bootstrapping replicates of ML analysis. When the gene phylogeny was not consistent with the species phylogeny (reviewed in Soltis et al. [2005]), we performed a tree topology test to see whether they are significantly different by using the Kishino–Hasegawa test (Kishino and Hasegawa 1989) and the Shimodaira–Hasegawa test (Shimodaira and Hasegawa 2001) implemented in the software TREE-PUZZLE (Schmidt et al. 2002). Taxa with multiple ancient WG duplication events in their lineage were not chosen to minimize the effects of gene duplication events on the topology test.

### Analysis of Asymmetric Sequence Rate Evolution and Positive Selection

After the identification of orthologs, the rate of sequence evolution was computed using the software Codeml in PAML 4 (Yang 2007). We followed the analytical procedure described in Blanc and Wolfe (2004). Two hypotheses, unconstrained rate of evolution (i.e., asymmetric sequence evolution) and clock-like rate of evolution (i.e., symmetric sequence evolution), were tested using an ML approach with the JTT (Jones, Taylor, and Thorton) matrix for the correction of multiple substitutions. To detect which model fits better with our data, a likelihood ratio test (LRT) was applied. The twice likelihood ratio (2*δL*) was compared against a chi-square distribution with df = 1 following the equation: *2δL* = −2(*Ln1* *−* *Ln2*), where *Ln1* is the likelihood estimates from the first test, and *Ln2* is the likelihood estimates from the second test. Adjusted *P* values (i.e., *Q* value < 0.05) were applied to correct for multiple testing using the false discovery rate (FDR) method (Storey and Tibshirani 2003). To test whether duplicated pairs with subcellular relocalization are often associated with asymmetric sequence rate evolution, the Fisher’s exact test and Monte Carlo randomization tests were applied. For the Monte Carlo randomization test, we specified a test statistic (DIF) as a measure of the absolute difference in the frequency of asymmetric sequence evolution of duplicated pairs with different subcellular localization and those with same subcellular localization. We then compared the DIF value from the observed data (DIF_obs_) against the null distribution of the simulated DIF value from 10,000 randomized data (DIF_sim_; as null hypothesis). If the null hypothesis is rejected, the frequency of asymmetric sequence evolution is significantly higher in duplicated pairs with different subcellular localization than those with same subcellular localization.

A positive selection test was performed on duplicated pairs that showed asymmetric sequence rate evolution using the software Codeml in PAML 4 (Yang 2007). A branch-site model and LRT were applied to test whether there is any positive selection acting on individual codons. Two LRTs were performed (Model A_test1_ and Model A_test2_) on each triplet (duplicate 1, duplicate 2, and their ortholog). The copy with accelerated sequence evolution was assigned as the foreground lineage, whereas the others were designated as the background lineage. To reduce false positives, only positively selected sites with >0.95 Bayesian posterior probability were considered using the Bayes Empirical Bayes (BEB) analysis.

To better quantify asymmetric sequence rate evolution in terms of nonsynomymous (d*N*) substitution rate and d*N*/d*S* ratio in the case studies, we implemented the LRT to examine the presence and absence of asymmetric d*N* and d*N*/d*S* between duplicates using the software HyPhy (Pond et al. 2004) and software PAML 4 (Yang 2007). In contrast to the PAML package, HyPhy allows users to examine the relative nonsynonymus (d*N*) substitution rate between any given two duplicated genes using ML (Pond et al. 2004). After determining the orthologs and tree topology, we followed the analytical procedures described in the HyPhyl manual (Pond et al. 2004) and the PAML 4 manual (Yang 2007) to examine the absence and presence of asymmetric d*N* and *ω* (=d*N*/d*S*) between duplicates. Briefly, when testing the evolutionary scenario that there has been asymmetric d*N* and d*N*/d*S* evolution between duplicated genes (e.g., d*N_1_* and *ω_1_* in gene 1 vs. d*N_2_* and *ω_2_* in gene 2), the null hypothesis of d*N_1_* (or *ω_1_*) = d*N_2_* (or *ω_2_*) versus the alternative hypothesis of d*N_1_* (or *ω_1_*) ≠ d*N_2_* (or *ω_2_*) was evaluated using the LRT. If the log-likelihood value of the alternative hypothesis is significantly higher than that of the null hypothesis, it suggests that there has been asymmetric d*N* evolution between gene 1 and gene 2.

### Analysis of Protein Isoelectric Points

We examined whether there is any significant difference in protein isoelectric point (pI) between duplicate pairs with and pairs without subcellular relocalization. The pI values of the *Arabidopsis* peptide sequences were obtained using the Protein Isoelectric Point of the Sequence Manipulation Suite database with *p*K values from EMBOSS. A *t*-test was used to examine whether there is any difference in pI values between duplicates with and without subcellular relocalization. All statistical analyses were performed using the statistical package R.

## Results and Discussion

### Diverged Localization of Two Duplicated Peroxidases, Accelerated Sequence Evolution, and Regulatory Neofunctionalization

**Fig. 1.—:**
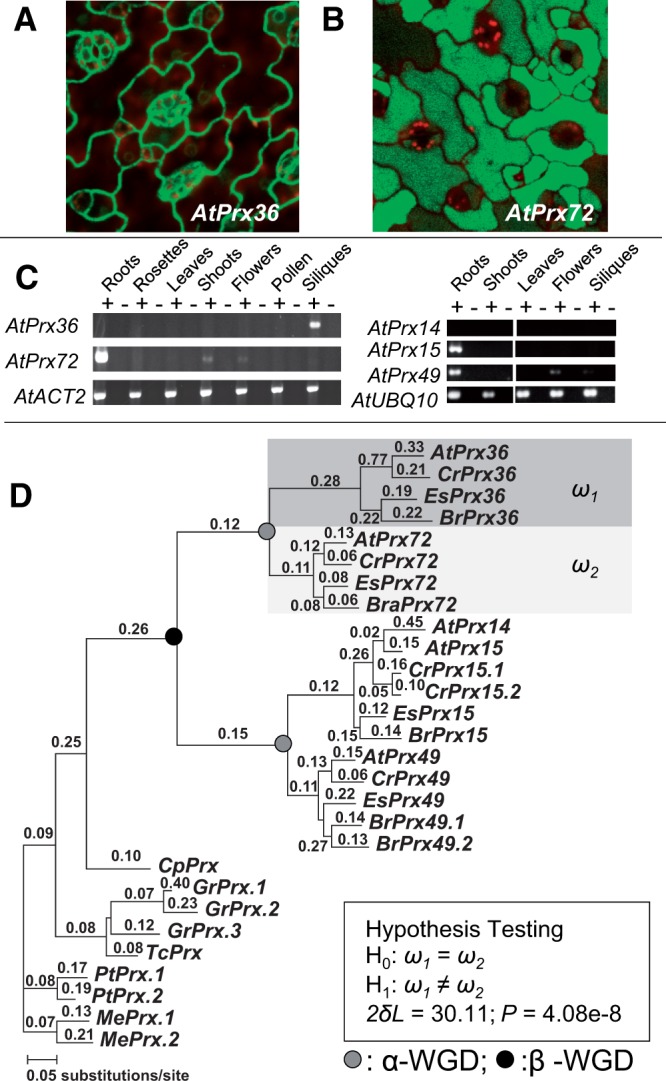
Subcellular relocalization, asymmetric sequence evolution, and gene expression divergence in a pair of peroxidases. (*A*) GFP subcellular localization of PRX36. (*B*) GFP subcellular localization of PRX72. (*C*) RT-PCR expression assays of *PRX36* and *PRX72*. Plus signs (+) indicate reactions with reverse transcriptase and minus signs (−) indicate reactions without reverse transcriptase. *ACT2* and *UBQ10* were positive controls. (*D*) PAML analysis of *PRX36, PRX72*, and their orthologs in other species. Numbers above the branches indicate the d*N/*d*S* ratios. d*N* analysis is shown in supplementary figure S4, Supplementary Material online. Species include: At, *A. thaliana*; Cr, *Capsella rubella*; Es, *Eutrema salsugineum*; Br, *Brassica rapa*; Cp, *C. papaya*; Gr, *G. raimondii*; Tc, *T. cacao*; Pt, *P. trichocarpa*; and Me, *M. esculenta*. See supplementary figures S2 and S4, Supplementary Material online, for locus numbers of each gene.

During a study of duplicated gene expression in *A. thaliana* (Liu et al. 2011), we identified two class III peroxidase genes with organ-specific complementary expression patterns. The genes, *PRX36* (At3g50990) and *PRX72* (At5g66390), were derived from the alpha polyploidy event at the base of the Brassicaceae family (Blanc et al. 2003; Bowers et al. 2003). They code for proteins with divergent N-termini (supplementary fig. S2*A*, Supplementary Material online). The divergent N-terminus is the result of the abolishment of the original start codon and gain of a new start codon (supplementary fig. S2*B*, Supplementary Material online). As localization signals are often located at the N-terminus, we hypothesized that they might have different subcellular localizations. *PRX36* (also known as *PER36*) recently was shown to localize to the cell wall (Kunieda et al. 2013). We concurrently performed a GFP subcellular localization assay to determine whether the products of both *PRX36* and *PRX72* are targeted to different subcellular locations (see Materials and Methods for details). We found that PRX36 is localized to the cell wall (fig. 1*A*) whereas PRX72 is located in the cytosol (fig. 1*B*). It has been shown that the cell wall localization signal peptide can be located at the N-terminus (e.g., Chen et al. 2004) and thus the divergent N-terminus of PRX36 (supplementary fig. S2, Supplementary Material online) may function as a cell wall localization signal peptide. Our data indicate that there has been a change in localization after gene duplication, and suggest that PRX36 was relocalized.

*PRX36* and *PRX72* have contrasting expression patterns. From both microarray expression data (from Schmid et al. 2005) and our RT-PCR assays, *PRX36* and *PRX72* showed a complementary and reciprocal expression pattern, where only *PRX36* is expressed in siliques and only *PRX72* is expressed in roots (fig. 1*C* and supplementary fig. S3*A*, Supplementary Material online). To gain insights into the ancestral expression pattern, we examined expression of three peroxidase genes that are related to *PRX36* and *PRX72* by the beta-WG duplication event in the Brassicaceae lineage: *PRX49 (*At4g36430) and the tandem duplicate pair of *PRX14* (At2g18140) and *PRX15* (At2g18150); tree topology testing indicated that all three genes share a common ancestor with *PRX36* and *PRX72* after the divergence of the Brassicaceae from the Caricacaceae (fig. 1*D* and supplementary fig. S4*A*, Supplementary Material online). Our RT-PCR assays showed that *PRX49* and *PRX15* are highly expressed in roots with low or no expression in most other organ types (fig. 1*C*), largely consistent with the microarray results (supplementary fig. S3*A*, Supplementary Material online). In contrast, *PRX14* was not expressed in any of the assayed organ types (fig. 1*C*), although the microarray results showed expression in roots (supplementary fig. S3*A*, Supplementary Material online). None of the three genes are expressed in siliques, as is *PRX36*. Their expression patterns are much more similar to *PRX72* than *PRX36*, suggesting that *PRX36* has a derived expression pattern of high expression in siliques and no expression in roots which is indicative of regulatory neofunctionalization (a new expression pattern) of *PRX36* after its formation by gene duplication.

Duplicated genes sometimes show an asymmetric rate of amino acid sequence evolution with one copy evolving more rapidly than the other, which has been associated with functional divergence and neofunctionalization (e.g., Blanc and Wolfe 2004; Liu and Adams 2010; Panchin et al. 2010; Owens et al. 2013). To determine whether *PRX36* shows accelerated and asymmetric sequence rate evolution, we performed a detailed sequence rate analysis by incorporating orthologous sequences from outgroup species in a phylogenetic framework using analyses of d*N*/d*S* and d*N* (see Materials and Methods for more details). The results showed that *PRX36* evolved much faster than *PRX72* after gene duplication (fig. 1*D* and supplementary fig. S4*B*, Supplementary Material online).

### Identification of Brassicaceae-Specific Duplicates with Divergent Localization

After characterizing a case of subcellular relocalization of a pair of duplicated peroxidase genes, we assembled a set of gene pairs in *A. thaliana* that were formed by duplication during the evolution of the Brassicaceae family, for which experimental localization data are available for both duplicates. We used experimental localization data because results from localization prediction programs often show inconsistency among programs and high error rates (e.g., Heazlewood et al. 2005; Millar et al. 2006). We used the GFP data from the SUB-cellular location database for *Arabidopsis* proteins (SUBA) database (Heazlewood et al. 2005) plus results from a literature search for papers on fluorescent protein localization experiments published since the last update of SUBA (see Materials and Methods for details). For a set of evolutionarily recent duplicates, we included genes duplicated by the α-WG duplication event (α-WGD) at the base of the Brassicaceae family (Blanc et al. 2003; Bowers et al. 2003) plus tandem duplicates (Haberer et al. 2004) and other types of duplicates that originated during the evolution of the Brassicaceae family (see Materials and Methods for details). We obtained a final set of 128 gene pairs (supplementary table S3, Supplementary Material online).

We found that 19 of 128 duplicate pairs (15%) showed divergent localizations (table 1 and supplementary table S3, Supplementary Material online). Fourteen of them showed complete changes of protein subcellular localization, whereas five of them showed expansion or contraction of protein subcellular localization. Use of GFP experimental data resulted in a relatively small set of duplicated gene pairs (128), from a genome-wide perspective, which makes it more difficult to infer a percentage of relocalized duplicates, but provided an increased confidence in localization inferences compared with localization prediction programs. Although there is no evidence to suggest that the gene pairs in our study are a biased set in terms of conserved versus diverged subcellular localization, such a bias is possible from a relatively small sample of duplicated genes that could result in an underestimate or overestimate of the percentage with subcellular relocalization. Nonetheless our results suggest that a considerable number of duplicates may have evolved different subcellular localizations during the evolution of the Brassicaceae family. A recent study using a prediction program to infer subcellular relocalization of duplicated genes in *Arabidopsis* and several other eukaryotes inferred that 28% of duplicates in *A. thaliana* have been relocalized (Byun and Singh 2013). Our estimation of 15% may be lower because we used experimental data rather than a subcellular localization prediction program, we only analyzed genes formed by duplication during the evolution of the Brassicaceae, and our sample size was smaller. A study of subcellular relocalization in *Saccharomyces cerevisiae*, using GFP data, showed that 88 of 238 duplicate protein pairs (37%) showed different localization. Thus subcellular relocalization of duplicated genes may be relatively common in other organisms too.

**Table 1:** Subcellular Localization, Type of Gene Duplication, Sequence Rate Evolution, and Putative Gene Function of 19 Relocalized Duplicated Pairs

Gene 1	SCL	Gene 2	SCL	Duplicate Type	Putative Function	Reference
At3g49560	cp	At5g24650	mt; cp	α-WGD	Inner membrane translocase	Murcha et al. 2007
(*TIM*)		(*TIC/M*)				
**At2g23800**	ER	At4g36810	cp	α-WGD	Geranylgeranyl pyrophosphate synthase	Okada et al. 2000
**(*GGPS2*)**		(*GGPS1*)				
At1g14450	ER	At2g02510	mt; per; cp	α-WGD	NADH dehydrogenase	Abu-Abied et al. 2009
(*NADH*)		(*NADH*)				
At1g17050	cp	At1g78510	ER	α-WGD	Solanesyl diphosphate synthase	Jun et al. 2004; Hirooka et al. 2005
(*SPS2*)		(*SPS1*)				
**At1g02510**	pm	At4g01840	Va	α-WGD	K^+^ channel protein	Becker et al. 2004; Voelker et al. 2006
**(*TPK4*)**		(*TPK5*)				
**At3g05790**	mt; cp	At5g26860	mt	α-WGD	Lon protease-like protein	Ostersetzer et al. 2007
**(*LON4*)**		(*LON1*)				
At1g55920	cp	At3g13110	mt	α-WGD	Serine *O*-acetyltransferase	Noji et al. 1998
(*SAT1*)		(*SAT3*)				
At3g01330	cy; nu	At5g14960	nu	α-WGD	E2F-like transcription factor	Kosugi and Ohashi 2002
(*DEL3*)		(*DEL2*)				
At1g13270	cp	**At3g25740**	mt; cp	α-WGD	Methionine aminopeptidase	Giglione et al. 2000
(*MAP1C*)		**(*MAP1B*)**				
At1g13460	per	At3g26020	nu; cy	α-WGD	The B subunit of protein phosphatase 2A	Matre et al. 2009
(*PP2A B' θ*)		(*PP2A B' η*)				
**At3g50990**	cw	At5g66390	cy	α-WGD	Class III peroxidase	This study
**(*PRX36*)**		(*PRX72*)				
At5g04870	per; lb	At3g10660	ER	α-WGD	Calcium-dependent protein kinase	Lu and Hrabak 2002; Dammann et al. 2003; Coca and San Segundo 2010
(*CPK1*)		(*CPK2*)				
At2g39800	cyb	At3g55610	cy	α-WGD	△-1-pyrroline-5-carboxylate synthetase	Székely et al. 2008
(*P5CS1*)		(*P5CS2*)				
At3g10550	cyb	**At5g04540**	cy	α-WGD	Myotubularin-like phosphatases	Ding et al. 2012
(*MTM1*)		**(*MTM2*)**				
At1g31630	cy	At1g31640	nu	Tandem	MADS-box transcription factor	Carrie et al. 2009
(*AGL86*)		(*AGL92*)				
**At2g33110**	ER	At2g33120	pm; en	Tandem	SNARE binding protein	Uemura et al. 2004
**(*VAMP723*)**		(*VAMP722*)				
At3g08720	nu	At3g08730	cy	Tandem	Serine/threonine protein kinase	Mahfouz et al. 2006
(*S6K2*)		(*S6K1*)				
At5g39510	TGN/PVC	**At5g39630**	ER	Tandem	SNARE binding protein	Uemura et al. 2004
(*VTI11*)		**(*VTI14*)**				
At4g15415	nu; cy	At3g21650	mt; cy	Other	The B subunit of protein phosphatase 2A	Matre et al. 2009
(*PP2A B' γ*)		(*PP2A B' ζ*)				

Note.—cp, chloroplast; cpl, cell plate; cy, cytosol; cyb, cytosolic subcellular body; ck, cytoskeleton; en, endosome; lb, lipid bodies; mt, mitochondrion; nu, nucleus; per, peroxisome; pm, plasma membrane; SCL, subcellular localization; TGN/PVC, trans-Golgi network/prevacuolar compartment; va, vacuole. Boldface indicates the statistically significant accelerated copy after correction for multiple testing.

### Duplicates with Divergent Localization Often Have Asymmetric Sequence Evolution

**Fig. 2.—:**
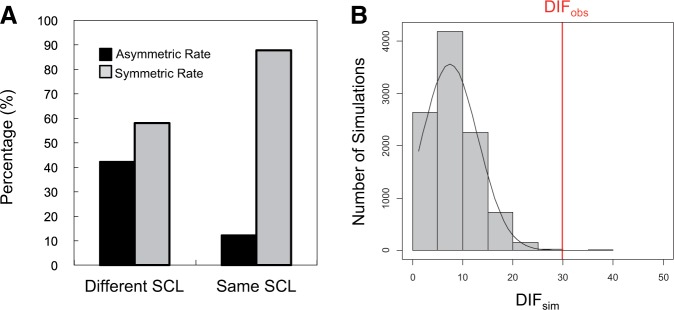
Asymmetric sequence evolution in duplicated gene pairs. (*A*) Diagram showing the frequency of asymmetrically evolved duplicated pairs in duplicated pairs with different subcellular localization (SCL) and duplicated pairs with the same SCL. (*B*) Histogram showing the distribution of the difference in frequency of asymmetric sequence evolution between duplicated pairs with different SCL and those with the same SCL (DIF_sim_) from 10,000 Monte Carlo randomization tests. Red line indicates the observed value (DIF_obs_).

After relocalization, a duplicated gene could perform a similar function, or there could be functional diversification compared with the ancestral function. Asymmetric sequence rate evolution, where one copy has experienced a significantly elevated rate of amino acid changes relative to the other copy, can be used as an indicator for possible functional divergence (e.g., Dermitzakis and Clark 2001; Blanc and Wolfe 2004; Kim and Yi 2006; Byrne and Wolfe 2007). To evaluate whether there has been any significant asymmetric sequence evolution for the 19 duplicate pairs with divergent localization, and to test the hypothesis that relocalized duplicates show more cases of asymmetric sequence rate evolution than those with the same subcellular localization, we identified orthologs of each duplicate from outgroup species and performed asymmetric sequence rate analysis (e.g., Blanc and Wolfe 2004; Liu et al. 2011; see Materials and Methods for details). Based on our analysis, 8 of 19 relocalized duplicates (42%) and 13 of 109 nonrelocalized duplicates (12%) showed significant asymmetric sequence evolution (table 1; LRT: *Q* < 0.05 in supplementary table S3, Supplementary Material online; false discovery rate-corrected for multiple tests). The protein sequences of duplicated pairs with subcellular relocalization evolve asymmetrically more frequently than those without subcellular relocalization (Fisher’s exact test: One-tailed *P* = 4 × 10^–^^3^; Monte Carlo randomization test: Two-tailed *P* = 9 × 10^–^^4^; fig. 2). We also compared the frequency of relocalized duplicate gene pairs with asymmetric sequence evolution obtained here with gene pairs from Blanc and Wolfe (2004), who analyzed 833 duplicated gene pairs from the α-WGD using the same method to what we used. A randomization test using results from Blanc and Wolfe (2004) showed that the percentage of gene pairs with asymmetric rate evolution was significantly lower than for the relocalized gene duplicates studied here (*P* = 0.038).

### Protein Subcellular Relocalization Is Not Associated with Changes in Protein Isoelectric Point

**Fig. 3.—:**
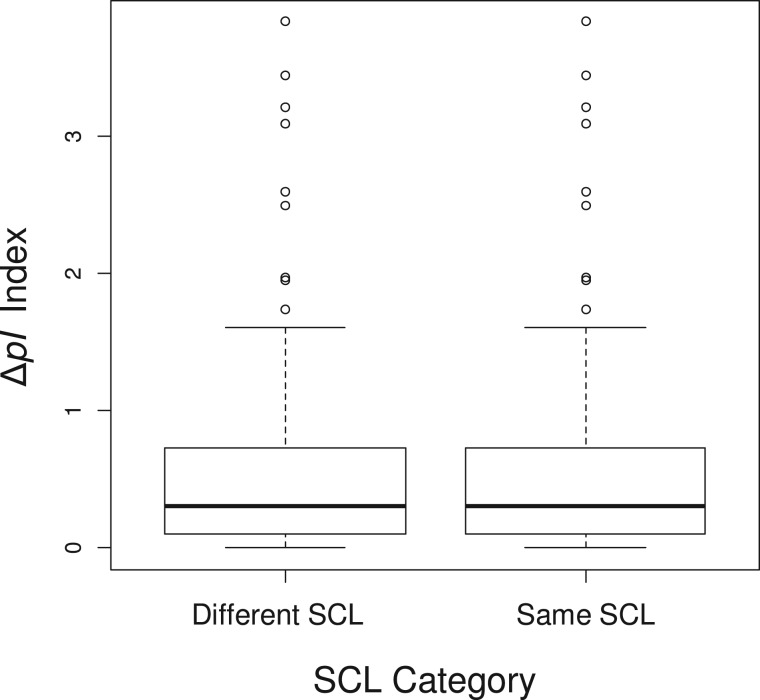
Analysis of the pI difference (ΔpI) between duplicated pairs. Box plots of ΔpI in gene pairs with different subcellular localization (SCL) and those with the same subcellular localization. Higher ΔpI values indicate a greater difference in pI between the duplicates.

After relocalization, there can be a change in the protein isoelectric point (pI), for adapting to the pH of the new cellular compartment, as shown in yeast (Marques et al. 2008). Thus, we might expect to find a greater pI difference for relocalized duplicated genes than nonrelocalized duplicated genes because relocalized duplicated genes would evolve a distinct pI through subcellular pH adaptation. To test the hypothesis that pI changes occur in relocalized duplicates in plants, we estimated the pI difference (ΔpI) between duplicated pairs. Our analysis showed no significant difference between duplicated pairs with subcellular localization and those without subcellular localization (fig. 3; *t*-test: two-tailed *P* = 1.0000). Thus it appears that yeast and plants differ in this regard, although we cannot eliminate the possibility that our smaller sample size (128 vs. 238) might have an effect.

### Case Studies of Relocalized Genes

We next present sequence and expression analyses to further characterize five cases of subcellular relocalization. The goals were to infer which gene in a duplicate pair has been relocalized, further characterize sequence rate evolution in the relocalized genes, determine whether the relocalized genes show changes in expression patterns, and infer sequence changes that may have led to relocalization in some cases with sufficient available localization data. The five duplicated gene pairs selected for analyses were members of families for which there were enough localization data available so that we could make an inference about which gene was relocalized.

### Neolocalization and Positive Selection in *VAMP723*

**Fig. 4.—:**
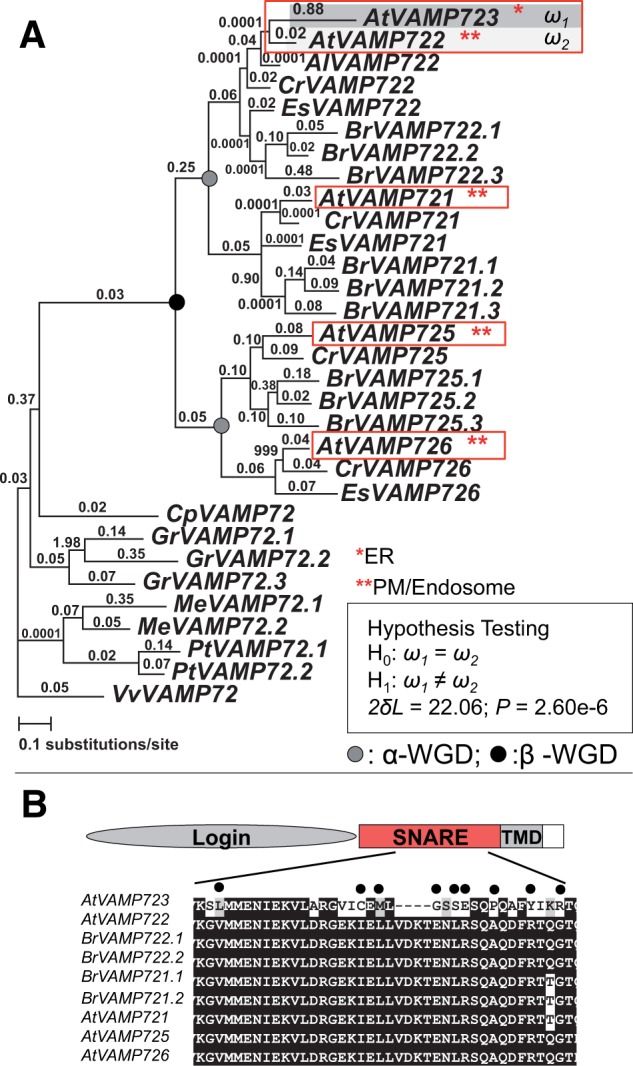
Neolocalization, asymmetric sequence evolution, and gene expression divergence in a pair of *VAMP* proteins. (*A*) PAML analysis of *VAMP* genes. Numbers above the branches indicate the d*N/*d*S* ratios. d*N* analysis is shown in supplementary figure S5, Supplementary Material online. Subcellular localization for the proteins in *A. thaliana* is highlighted in red. *VAMP722* is relocalized from PM/endosome to ER. Species include: At, *A. thaliana*; Al, *Arabidopsis lyrata*; Cr, *Capsella rubella*; Es, *Eutrema salsugineum*; Br, *Brassica rapa*; Cp, *C. papaya*; Gr, *G. raimondii*; Tc, *T. cacao*; Pt, *P. trichocarpa*; Me, *M. esculenta*; and Vv, *V. vinifera.* See supplementary figure S5, Supplementary Material online, for locus numbers of each gene. (*B*) Amino acid alignment showing the position of positively selected sites in the SNARE domain of *VAMP723* inferred using the empirical Bayes approach.

One gene pair showing accelerated sequence rate evolution of one copy is the tandemly duplicated gene pair for vesicle-associated membrane proteins *VAMP723* (At2g33110) and *VAMP722* (At2g33120) that contain SNARE (soluble *N*-ethylmaleimide sensitive factor receptors) domains (Uemura et al. 2004; Sanderfoot 2007). *VAMP722* has two major functions: Involvement in secretory trafficking to the plasma membrane and cell plate formation (Zhang et al. 2011), and contributing to the plant immune response upon the infection with powdery mildew fungi by participating in the formation of an SDS-SNARE complex with the plasma membrane proteins *PEN1* and *SNAP33* (Kwon et al. 2008); in contrast the function of *VAMP723* has not been characterized. We used a gene family approach and GFP subcellular localization data from a study of 54 genes with SNARE domains (Uemura et al. 2004) to infer the ancestral, preduplication, state of localization. We found that there have been multiple duplications of the *VAMP72* genes to create five genes in *A. thaliana* (fig. 4 and supplementary fig. S5, Supplementary Material online). Only *VAMP723* is localized to the endoplasmic reticulum (ER), whereas the other four gene products are localized to the plasma membrane and endosome. These results strongly suggest that the ancestral state of localization for the *VAMP722/VAMP723* gene pair was to the plasma membrane and endosome, and that *VAMP723* has been relocalized to the ER. The relocalization appears to be a relatively recent evolutionary event. Results from our phylogenetic analysis showed that *VAMP723* and *VAMP722* phylogenetically group together, to the exclusion of the *VAMP722* genes from *Brassica* and *Eutrema* (fig. 4*A*), indicating that they are duplicates that formed during evolution of the *Arabidopsis* lineage after it diverged from the *Eutrema* lineage.

We investigated the sequence evolution of *VAMP723* and *VAMP722* in more detail and determined whether *VAMP723* showed any evidence of positive selection. We estimated the nonsynonymous substitution rate (d*N*) and the d*N*/d*S* (omega) ratio (*ω*) for the branch leading to members of the *VAMP72* subfamily in *A. thaliana*, as well as orthologs from *Brassica rapa*, *C. papaya*, *Gossypium ramondii*, *T. cacao*, *M. esculenta*, *P. trichocarpa*, and *V. vinifera*. The results indicated that *VAMP723* has a much higher d*N* and *ω* than its duplicated partner, *VAMP722* (fig. 3*A* and supplementary fig. S5, Supplementary Material online). From the positive selection analysis, several codons in *VAMP723* were detected as positively selected and the majority of those sites are located within the SNARE domain (table 2; fig. 4*B*).

**Table 2:** Parameter Estimates and LRT Statistics for the Gene *AtVAMP723*

Branch-Site Model	*P*	*L*	Estimates of Parameters	Positively Selected Sites
Model A test1	64	−6,306.97	*p_0_* = 0.733, *p_1_* = 0.036, (*p_2_* + *p_3_* = **0.231**)	144L (0.960^a^)
			*ω_0_* = 0.065, *ω_1_* = 1.000 , *ω_2_* = **6.229**	159C (1.000)
				161M (0.995)
				163G (0.996)
				165S (0.999)
				166E (1.000)
				169P (0.954)
				173Y (1.000)
				176R (0.951)
Model A test2	63	−6,311.76	*p_0_* = 0.391, *p_1_* = 0.019, (*p_2_* + *p_3_* = 0.590)	Not allowed.
			*ω_0_* = 0.064, *ω_1_* = 1.000 , *ω_2_* = 1.000	

Note.—*P*, number of free parameters for the estimation of ω ratios; *p_0__–__4_*, four different site classes in the branch-site model; *ω_0__–__2_*, three different *ω* ratios in four different site classes; *L*, the estimation of log-likelihood value. Boldface indicates parameters for positive selection.

^a^Value indicates the posterior probability based on the BEB analysis.

To determine whether *VAMP723* evolved a different expression pattern compared with its duplicated partner, *VAMP722,* and their closely related paralog *VAMP721*, we performed gene-specific expression assays using RT-PCR. Results from RT-PCR showed that all three genes were expressed in all examined organ types, but the expression of *VAMP723* was very weak in pollen (supplementary fig. S5*C*, Supplementary Material online). Thus, there has been only limited expression divergence of *VAMP723* after duplication from *VAMP722*.

### Relocalization, Novel Expression Pattern, and Accelerated Sequence Evolution of *VTI14*

**Fig. 5.—:**
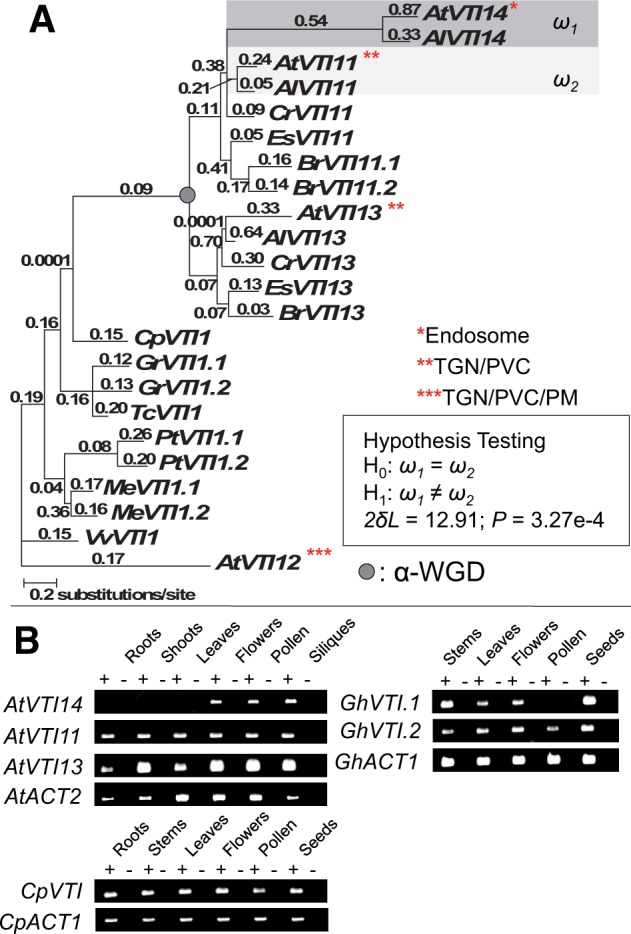
Neolocalization, asymmetric sequence evolution, and gene expression divergence in a pair of *VTI* proteins. (*A*) PAML analysis of *VTI* genes. Numbers above the branches indicate the d*N*/d*S* ratios. d*N* analysis is shown in supplementary figure S6, Supplementary Material online. Subcellular localization for the proteins in *A. thaliana* is highlighted in red. *VTI14* was relocalized from TGN/PVC (trans-Golgi network/prevacuolar compartments) to endosome. Species include: At, *A. thaliana*; Al, *Arabidopsis lyrata*; Cr, *Capsella rubella*; Es, *Eutrema salsugineum*; Br, *Brassica rapa*; Cp, *C. papaya*; Gr, *G. raimondii*; Tc, *T. cacao*; Pt, *P. trichocarpa*; Me, *M. esculenta*; and Vv, *V. vinifera.* See supplementary figure S6, Supplementary Material online, for locus numbers of each gene. (B) RT-PCR expression assays of *VTI14*, *VTI11*, and *VTI 13* in *A. thaliana*, and their orthologs in *C. papaya* and *G. hirsutum*. Plus signs (+) indicate reactions with reverse transcriptase and minus signs (−) indicate reactions without reverse transcriptase. *ACT2* was a positive control in *A. thaliana*, whereas *ACT1* was a positive control in *C. papaya* and *G. hirsutum*.

The *VTI14* (At5g39630) gene is a member of the SNARE family, like the *VAMP* genes in the previous section. Based on both phylogenetic tree and syntenic block analyses, *VTI14* was formed by tandem duplication of *VTI11* (At5g39510), and the *VTI14*/*VTI11* precursor was derived by the α-WGD along with *VTI13* (supplementary fig. S6, Supplementary Material online). *VTI11* and *VTI13* (At3g2900), as well as the more distant paralog *VTI12*, are localized in the prevacuolar compartments and the trans-Golgi network (Uemura et al. 2004). *VTI14*, in contrast, is localized to the endosome (Uemura et al. 2004). Our gene family phylogenetic analysis suggested that the most recent common ancestral subcellular localization of the *VTI1* subfamily is localized in the prevacuolar compartments and the trans-Golgi network (fig. 5*A*), which would indicate that *VTI14* has gained a new targeting ability to the endosome since formation by tandem duplication.

To further examine the asymmetric sequence evolution in *VTI14*, we conducted a more detailed asymmetric rate analysis between *VTI11* and *VTI14* by examining both the omega ratio and the d*N* rate with additional taxon sampling. The ML analysis showed that *VTI14* evolved much faster than its duplicated partner, *VTI11* (fig. 5*A* and supplementary fig. S6, Supplementary Material online), indicative of asymmetric rate evolution between *VTI14* and *VTI11*. No evidence of positively selected sites was detected in *VTI14*, suggesting that the rapidly evolving sequence was the result of relaxation of purifying selection.

We examined expression patterns of *VTI14* to compare with *VTI11*, the other related paralog *VTI13*, and the orthologs from *C. papaya* and *G. hirsutum*. Gene-specific gene expression was conducted using RT-PCR. *VTI14* expression was restricted to flowers, pollen, and siliques (fig. 5*B*). In contrast, orthologs from *Carica* and *Gossypium*, as well as both closely related paralogs, *VTI11* and *VTI13*, showed a broad expression pattern across multiple organ types (fig. 5*B*). Thus, *VTI14* appears to have acquired a very restricted expression pattern after duplication from the common ancestor with *VTI11*.

### Relocalization and Regulatory Neofunctionalization in Pollen of *TPK4*

A pair of tandem-pore potassium ion (K^+^) channel proteins, *TPK4* (At1g02510) and *TPK5* (At4g01840), which are α-WG duplicates, shows different subcellular localizations. *TPK4* plays a role in potassium homeostasis and membrane voltage control in the growing pollen tube (Becker et al. 2004), but the function of *TPK5* remains uncharacterized. *TPK5* and four other members of this gene family are localized to vacuoles (Becker et al. 2004). In contrast, *TPK4* is localized to the plasma membrane (Becker et al. 2004) and ER (Dunkel et al. 2008). The localization data for the TPK family strongly suggest that the plasma membrane and ER are the derived subcellular location for *TPK4*.

**Fig. 6.—:**
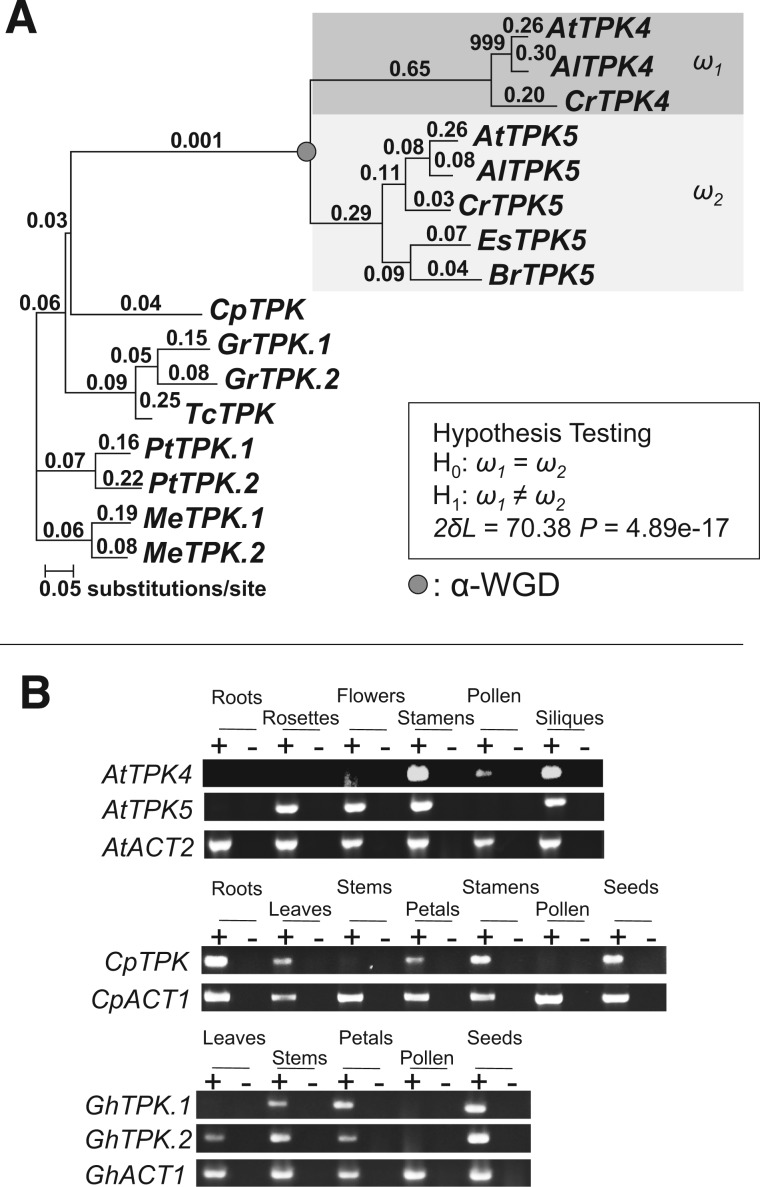
Asymmetric sequence evolution, neolocalization, and gene expression divergence in a pair of K^+^ channel protein proteins. (*A*) PAML analysis of *TPK* genes. d*N* analysis is shown in supplementary figure S7, Supplementary Material online. Numbers above the branches indicate the d*N*/d*S* ratios. Species include: At, *A. thaliana*; Al, *Arabidopsis lyrata*; Cr, *Capsella rubella*; Es, *Eutrema salsugineum*; Br, *Brassica rapa*; Cp, *C. papaya*; Gr, *G. raimondii*; Tc, *T. cacao*; Pt, *P. trichocarpa*; and Me, *M. esculenta.* See supplementary figure S7, Supplementary Material online, for locus numbers of each gene. (*B*) RT-PCR expression assays of *TPK4* and *TPK5* in *A. thaliana*, and their orthologs in *C. papaya* and *G. hirsutum*. Plus signs (+) indicate reactions with reverse transcriptase and minus signs (−) indicate reactions without reverse transcriptase. *ACT2* was a positive control in *A. thaliana*, whereas *ACT1* was a positive control in *C. papaya* and *G. hirsutum*.

Our sequence rate analyses of d*N*/d*S* and d*N* showed that *TPK4* evolved faster than its duplicate *TPK5* (fig. 6*A* and supplementary fig. S7, Supplementary Material online). The asymmetric rate evolution in both *ω* and d*N* indicated that *TPK4* has accumulated many more amino acid changes than *TPK5* since gene duplication.

*TPK4* is predominately expressed in pollen (Becker et al. 2004). Our RT-PCR assay indicated that *TPK5* is broadly expressed, in contrast to *TPK4* (fig. 6). Thus, the expression profile between *TPK5* and *TPK4* has diverged in a complementary organ-specific manner. The complementary organ-specific expression pattern between *TPK4* and *TPK5* can result from one of two possible evolutionary fates, regulatory subfunctionalization and regulatory neofunctionalization. We therefore conducted expression assays of their orthologs from closely related outgroup species in Eurosid II (i.e., *C. papaya* and *G. hirsutum*) by using RT-PCR. Orthologs from both *C. papaya* and *G. hirsutum* were not expressed in pollen (fig. 6*B*), suggesting that *TPK4* acquired its novel regulatory context in pollen (i.e., regulatory neofunctionalization). In contrast, *TPK5* still reflects its ancestral expression pattern with a broad expression profile except for pollen.

### Regulatory Neofunctionalization in Pollen and Potential Sequence Changes Causing Neolocalization of *CPK2*

**Fig. 7.—:**
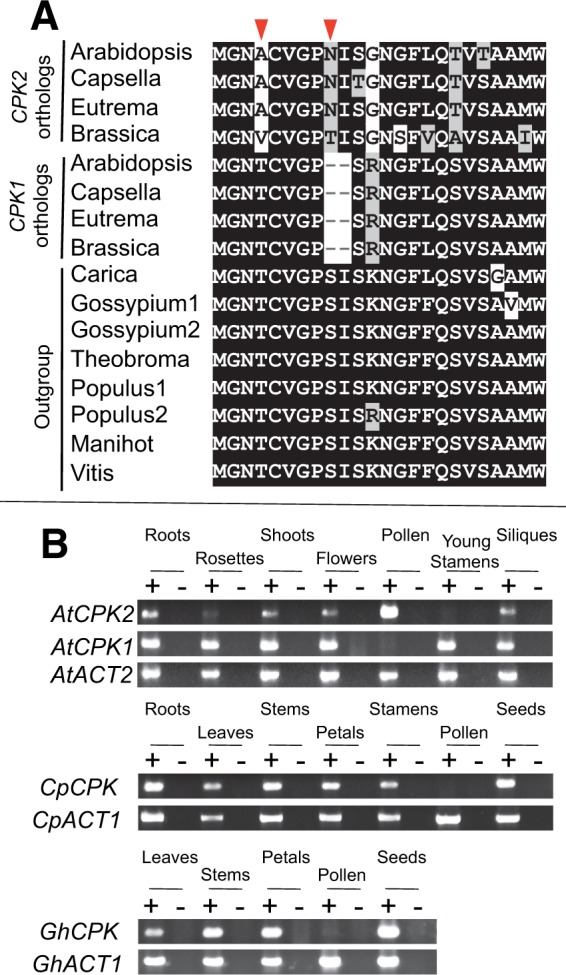
Neolocalization and gene expression divergence in *CPK2*. (*A*) Alignment showing the N-terminal targeting region. Arrowheads indicate *CPK2*-specific amino acid changes in the *CPK2* orthologs. Gene locus numbers are in supplementary figure S8, Supplementary Material online. (*B*) RT-PCR expression assays of *CPK1* and *CPK2* in *A. thaliana*, and their orthologs in *C. papaya* and *G. hirsutum*. Plus signs (+) indicate reactions with reverse transcriptase and minus signs (−) indicate reactions without reverse transcriptase. *ACT2* was a positive control in *A. thaliana*, whereas *ACT1* was a positive control in *C. papaya* and *G. hirsutum*.

To examine sequence changes that cause subcellular relocalization, and characterize expression and sequence evolution in another gene whose product has been relocalized, we studied a pair of calcium-dependent protein kinase genes, *CPK2* (At3g10660) and *CPK1* (At5g04870) that were duplicated by the α-WG duplication. GFP localization experiments have shown that they possess different subcellular localization ability: *CPK2* is localized in the ER (Lu and Hrabak 2002) and *CPK1* is localized in peroxisomes and lipid bodies (Dammann et al. 2003; Coca and San Segundo 2010). However, no comparisons of the localizations between the two genes have been made previously. Thus, we evaluated the previous localization studies to gain insights into which gene product was relocalized and what sequence changes may have allowed for relocalization. Lu and Hrabak (2002) experimentally showed that the first ten amino acids of *CPK2* are enough for its subcellular localization to the ER. Dammann et al. (2003) showed that replacing the first seven amino acids of *CPK1* resulted in loss of peroxisome targeting, indicating that the region is a type II N-terminal peroxisomal targeting signal (PTS2) (Reumann 2004). Thus, the peroxisome targeting function appears to be located in the first seven amino acids which we show are highly conserved in other species (fig. 7*A*). This implies that *CPK1* retains the ancestral subcellular localization and *CPK2* acquired its subcellular localization to the ER after duplication. It appears that mutations in amino acids 4 and/or 9 of *CPK2* may have allowed for targeting to the ER, and the substitution of thymine to alanine or valine at position 4 abolished targeting to the peroxisome (fig. 7*A*).

To determine whether there has been divergence in expression patterns of *CPK1* and *CPK2* since gene duplication, we compared their expression profiles across different organ types by analyzing microarray data from 63 different organ types and developmental stages (Schmid et al. 2005). *CPK2* is predominantly expressed in roots, stamens, and pollen, whereas *CPK1* is broadly expressed across multiple organ types but not in pollen (supplementary fig. S3*B*, Supplementary Material online). Thus, the expression profile between *CPK1* and *CPK2* diverged in a complementary organ-specific manner. To confirm the microarray data, we performed expression assays using RT-PCR. *CPK1* was strongly expressed in every examined organ type except for pollen where no expression was detected (fig. 7*B*). In contrast, *CPK2* was predominantly expressed in pollen, and showed weaker expression in roots, shoots, flowers, and siliques (fig. 7*B*). Thus, *CPK1* and *CPK2* show a complementary organ-specific expression pattern in pollen. We assayed expression of orthologs from *C. papaya* and *G. hirsutum* using RT-PCR with multiple organ types. Both species showed expression in various organ types but not in pollen (fig. 7*B*), strongly suggesting that *CPK1* reflects the ancestral expression pattern and the expression of *CPK2* in pollen was derived after gene duplication. Thus, *CPK2* has undergone regulatory neofunctionalization in pollen. Finally, asymmetric sequence evolution was not found between *CPK2* and *CPK1* (supplementary fig. S8, Supplementary Material online).

### Potential Sequence Changes Causing Neolocalization of *PP2A B*′θ

**Fig. 8.—:**
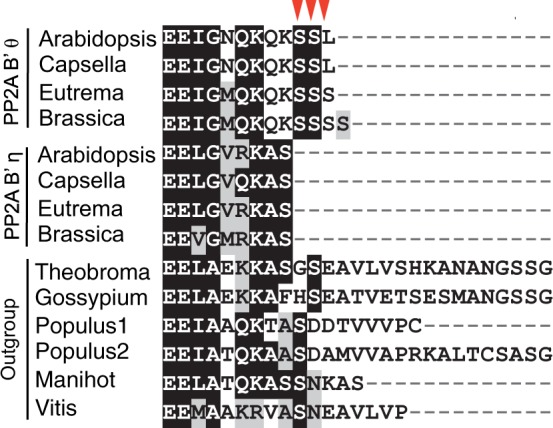
Alignment of subcellular targeting signal regions in *PP2A B'*. The C-terminal region of a pair of protein phosphatase 2A proteins, *PP2A B′θ* and *PP2A B′η*, and their orthologs in the outgroup species are shown. Signal peptides for peroxisome localization are indicated by inverted triangles. Gene locus numbers are in supplementary figure S10, Supplementary Material online.

A pair of genes for the beta subunit of phosphatase 2A *B′*, *PP2A B′θ* (At1g13460) and *PP2A B′η* (At3g26020), was formed by the α-WG duplication. GFP localization experiments revealed that *PP2A B′θ* localized to peroxisomes and *PP2A B′η* localized to the nucleus and cytosol (Matre et al. 2009). When the four amino acids at the C-terminus were deleted from *PP2A B′θ,* it no longer localized to the peroxisomes (Matre et al. 2009), indicating that the SSL peptide at the C-terminus, which is one of the PTS1 peroxisome localization signals (Reumann et al. 2007), is responsible for localization to the peroxisomes. The *PP2A B′* genes have not been studied in the context of their evolution after duplication. We performed comparative sequence analysis to determine which gene’s product shows relocalization. We compared the C-termini of *PP2A B′θ* and *PP2A B′η* with orthologs in outgroup species to gain insights into the ancestral localization (fig. 8). Only *PP2A B′θ* contains the SSL peroxisome localization peptide, strongly suggesting that the ancestral subcellular localization is not to peroxisomes. Thus it appears that *PP2A B′θ* has been relocalized to the peroxisomes by creation of an SL peptide at the C-terminus, either by addition of the SL peptide or by mutation of two amino acids after duplication that were independently deleted in *PP2A B′η* (fig. 8 and supplementary fig. S9*A*, Supplementary Material online). In addition, we predicted the subcellular localization of orthologs from outgroup species using the PTS1 peroxisome predictor software (Neuberger et al. 2003). None of them showed evidence of potential peroxisomal targeting (supplementary fig. S9*B*, Supplementary Material online), further supporting the inference that *PP2A B′θ* relocalized to peroxisomes after gene duplication. Additional support for neolocalization comes from the inference of their ancestral subcellular localization using a gene family approach. In the *PP2A B′* gene family, two additional members, At3g21650 (*PP2A B′ζ*) and At4g15414 (*PP2A B′γ*), have been shown to target mitochondria/cytosol and nucleus/cytosol (Matre et al. 2009), suggesting that peroxisomal targeting is not the ancestral state in the *PP2A B′* gene family.

The SSL motif is present in *PP2A B′θ* in *Arabidopsis* and *Capsella*, but not in *Brassica* or *Eutrema* that have SSS and SSSS, respectively (fig. 8). *Arabidopsis* and *Capsella* belong to the same tribe within the Brassicaceae, whereas *Brassica* and *Eutrema* are in a different clade (Bailey et al. 2006). Thus, it appears that relocalization of *PP2A B′θ* occurred in a common ancestor of *Arabidopsis* and *Capsella* after divergence from the common ancestor of *Brassica* and *Eutrema*. *PP2A B′θ* was formed by duplication during the α-WG duplication at the base of the Brassicaceae family. Thus, relocalization of *PP2A B′θ* in the *Arabidopsis*–*Capsella* lineage may have occurred long after its formation by gene duplication. An alternative possibility is that relocalization of *PP2A B′θ* occurred in a common ancestor of all four Brassicaceae species mentioned above and then *PP2A B′θ* in *Brassica* and *Eutrema* experienced mutations that abolished peroxisomal localization.

Except for marginally significant asymmetric *ω* evolution (supplementary fig. S10*A*, Supplementary Material online), asymmetric amino acid or d*N* sequence evolution was not found between *PP2A B′θ* and *PP2A B′η* (table 1; supplementary fig. S10*B*, Supplementary Material online). Finally, *PP2A B′θ* and *PP2A B′η* have very similar expression patterns, from AtGenExpress microarray data (Schmid et al. 2005).

### Features of Relocalized Genes

Asymmetric protein sequence rate evolution in one member of a duplicate pair has been inferred as a potential indicator of functional divergence because one copy has experienced an accelerated rate of amino acid replacements in comparison to its duplicated partner (e.g., Dermitzakis and Clark 2001; Blanc and Wolfe 2004; Kim and Yi 2006; Byrne and Wolfe 2007). In this study, we found that significantly more gene pairs with divergent localization of the products showed asymmetric rate evolution (42%) than those pairs whose products have the same localization location (12%). We then presented evidence for a few cases in which the more rapidly evolving gene has undergone relocalization. Some duplicated gene products that have undergone relocalization may have experienced changes in their cellular roles, or functional diversification, in the new cellular location, which were promoted by amino acid sequence changes. In duplicated genes of yeast, Marques et al. (2008) showed that gene pairs with the same subcellular localization tend to have lower amino acid sequence divergence than duplicated pairs with different subcellular localization. Our results are consistent with that study, indicating that the trend also extends to plants.

In this study, we show that duplicated genes with subcellular relocalization sometimes can show changes in expression patterns including regulatory neofunctionalization (a new expression pattern compared with the inferred ancestral state). In three cases (*CPK2, TPK4, PRX36*), we presented evidence that the gene with the relocalized product shows regulatory neofunctionalization. Another type of new expression pattern after duplication, the expression pattern becoming restricted to a small number of organ types compared with the ancestral gene, is illustrated by *VTI14.* The above examples indicate that duplicated genes whose products have been relocalized can show major changes in expression patterns and regulatory neofunctionalization. However, not all relocalized gene products show major changes in expression patterns, as shown with VAMP723 and *PP2A B′θ*. Subcellular relocalization is caused by changes in amino acid sequences, whereas regulatory neofunctionalization is typically caused by changes in regulatory sequences. Some genes in this study showed both phenomena (*CPK2, TPK4, VTI14*, and *PRX36*), whereas others showed only one.

In addition to asymmetric and accelerated sequence rate evolution and regulatory neofunctionalization, a few cases show evidence for changes in cellular roles or functions. For example, *PRX36* is specifically expressed in siliques and was shown recently to be required for seed coat mucilage extrusion (Kunieda et al. 2013). *PRX36* has been shown to loosen the outer cell wall of the seed coat in facilitating the mucilage extrusion by targeting to the outer cell wall. Its paralog, *PRX72,* is localized in the cytoplasm and thus not involved in cell wall functions. Thus, *PRX36* has a different cellular role, involved in seed coat mucilage extraction, than *PRX72*, but the products of both genes may function as peroxidases. In another example, involving *VTI11* and relocalized *VTI14*, *VTI11* has been shown to form SNARE complexes with *SYP2* and *SYP5*-type syntaxins (reviewed in Surpin and Raikhel 2004), which mediate trafficking to lytic vacuoles (Sanmartín et al. 2007). However, *VTI14* does not localize trans-Golgi network and the prevacuolar compartments but instead localizes to endosomes*.* Thus, *VTI14* likely has a different cellular role from *VTI11*.

### Evolutionary Timing of Subcellular Localization after Gene Duplication

In this study, we examined genes duplicated during the evolution of the Brassicaceae family as a group for assessing subcellular relocalization of evolutionarily recent duplicates in plants. A large majority of the duplicated genes showing subcellular relocalization were duplicated at the base of the Brassicacae in the α-WGD. A few cases of relocalization are more evolutionarily recent. For example, both *VAMP723* and *VTI14* were formed by duplication in the *Arabidopsis* lineage after it diverged from the *Brasssica* lineage. In contrast to our findings in plants, studies of subcellular relocalization showed that relocalization after gene duplication in animals and yeasts often evolved in duplicated genes that were derived from ancient WG duplication event in yeasts (∼100 Ma) and animals (∼500 Ma), but it was less common in younger duplicated genes (Marques et al. 2008; Kassahn et al. 2009; Szklarczyk and Huynen 2009). Both Szklarczyk and Huynen (2009) and Wang et al. (2009), in studies of mitochondrial protein localization, showed that relocalized duplicates were ancient, before the divergence of bilateria and before the divergence of vertebrates (Szklarczyk and Huynen 2009) or roughly corresponding to the time of the two WG duplication events early in animal evolution (Wang et al. 2009). Thus, it is possible that subcellular relocalization of duplicated genes is a more common ongoing process in plants than in animals and yeasts.

## Supplementary Material

Supplementary figures S1–S10 and tables S1–S4 are available at *Genome Biology and Evolution* online (http://www.gbe.oxfordjournals.org/).

Supplementary Data

## References

[evu191-B1] Abu-Abied M (2009). Identification of an *Arabidopsis* unknown small membrane protein targeted to mitochondria, chloroplasts, and peroxisomes. Protoplasma.

[evu191-B2] Adams KL, Daley DO, Whelan J, Palmer JD (2002). Genes for two mitochondrial ribosomal proteins in flowering plants are derived from their chloroplast or cytosolic counterparts. Plant Cell.

[evu191-B3] Alinsug MV, Yu CW, Wu K (2009). Phylogenetic analysis, subcellular localization, and expression patterns of *RPD3*/*HDA1* family histone deacetylases in plants. BMC Plant Biol..

[evu191-B4] Audemard E, Schiex T, Faraut T (2012). Detecting long tandem duplications in genomic sequences. BMC Bioinformatics.

[evu191-B5] Bailey CD (2006). Toward a global phylogeny of the Brassicaceae. Mol Biol Evol..

[evu191-B6] Barker MS, Vogel H, Schranz ME (2009). Paleopolyploidy in the Brassicales: analyses of the *Cleome* transcriptome elucidate the history of genome duplications in *Arabidopsis* and other Brassicales. Genome Biol Evol..

[evu191-B7] Becker D (2004). AtTPK4, an *Arabidopsis* tandem-pore K^+^ channel, poised to control the pollen membrane voltage in a pH^-^ and Ca^2+^-dependent manner. Proc Natl Acad Sci U S A..

[evu191-B8] Blanc G, Hokamp K, Wolfe KH (2003). A recent polyploidy superimposed on older large-scale duplications in the *Arabidopsis* genome. Genome Res..

[evu191-B9] Blanc G, Wolfe KH (2004). Widespread paleopolyploidy in model plant species inferred from age distributions of duplicate genes. Plant Cell.

[evu191-B10] Bowers JE, Chapman BA, Rong J, Paterson AH (2003). Unravelling angiosperm genome evolution by phylogenetic analysis of chromosomal duplication events. Nature.

[evu191-B11] Byrne KP, Wolfe KH (2007). Consistent patterns of rate asymmetry and gene loss indicate widespread neofunctionalization of yeast genes after whole-genome duplication. Genetics.

[evu191-B12] Byun SA, Singh S (2013). Protein subcellular relocalization increases the retention of eukaryotic duplicate genes. Genome Biol Evol..

[evu191-B13] Byun-McKay SA, Geeta R (2008). Protein subcellular relocalization: a new perspective on the origin of novel genes. Trends Ecol Evol..

[evu191-B14] Byun-McKay SA, Geeta R, Duggan R, Carroll B, McKay SJ, Pontarotti P (2009). Missing the subcellular target: a mechanism of eukaryotic gene evolution. Evolutionary biology: concept, modeling, and application.

[evu191-B15] Carrie C (2009). Approaches to defining dual-targeted proteins in *Arabidopsis*. Plant J..

[evu191-B17] Chen MH, Huang LF, Li HM, Chen YR, Yu SM (2004). Signal peptide-dependent targeting of a rice alpha-amylase and cargo proteins to plastids and extracellular compartments of plant cells. Plant Physiol..

[evu191-B18] Chong YT (2010). Characterization of the *Arabidopsis thaliana* exocyst complex gene families by phylogenetic, expression profiling, and subcellular localization studies. New Phytol..

[evu191-B19] Clough SJ, Bent AF (1998). Floral dip: a simplified method for *Agrobacterium*-mediated transformation of *Arabidopsis thaliana*. Plant J..

[evu191-B20] Coca M, San Segundo B (2010). *AtCPK1* calcium-dependent protein kinase mediates pathogen resistance in *Arabidopsis*. Plant J..

[evu191-B21] Dammann C (2003). Subcellular targeting of nine calcium-dependent protein kinase isoforms from *Arabidopsis*. Plant Physiol..

[evu191-B22] Dermitzakis ET, Clark AG (2001). Differential selection after duplication in mammalian developmental genes. Mol Biol Evol..

[evu191-B23] Devoto A (1999). Topology, subcellular localization, and sequence diversity of the *Mlo* family in plants. J Biol Chem..

[evu191-B24] Ding Y (2012). Divergent functions of the myotubularin (*MTM*) homologs *AtMTM1* and *AtMTM2* in *Arabidopsis thaliana*: evolution of the plant *MTM* family. Plant J..

[evu191-B25] Dixon DP, Hawkins T, Hussey PJ, Edwards R (2009). Enzyme activities and subcellular localization of members of the *Arabidopsis* glutathione transferase superfamily. J Exp Bot..

[evu191-B26] Dunkel M (2008). Targeting of vacuolar membrane localized members of the *TPK* channel family. Mol Plant..

[evu191-B27] Edgar CE (2004). MUSCLE: multiple sequence alignment with high accuracy and high throughput. Nucleic Acids Res..

[evu191-B28] Fawcett J, Van de Peer Y (2010). Angiosperm polyploids and their road to evolutionary success. Trends Evol Biol..

[evu191-B29] Flagel LE, Wendel JF (2009). Gene duplication and evolutionary novelty in plants. New Phytol..

[evu191-B30] Force A (1999). Preservation of duplicate genes by complementary, degenerative mutations. Genetics.

[evu191-B31] Giglione C, Serero A, Pierre M, Boisson B, Meinnel T (2000). Identification of eukaryotic peptide deformylases reveals universality of N-terminal protein processing mechanisms. EMBO J..

[evu191-B32] Haberer G, Hindemitt T, Meyers BC, Mayer KFX (2004). Transcriptional similarities, dissimilarities, and conservation of cis-elemetns in duplicated genes of *Arabidopsis*. Plant Physiol..

[evu191-B33] Heazlewood JL, Tonti-Filippini J, Verboom RE, Millar AH (2005). Combining experimental and predicted datasets for determination of the subcellular location of proteins in *Arabidopsis*. Plant Physiol..

[evu191-B34] Heazlewood JL, Verboom RE, Tonti-Filippini J, Small I, Millar AH (2007). SUBA: the *Arabidopsis* subcellular database. Nucleic Acids Res..

[evu191-B35] Heilmann I, Pidkowich MS, Girke T, Shanklin J (2004). Switching desaturase enzyme specificity by alternate subcellular targeting. Proc Natl Acad Sci U S A..

[evu191-B36] Hirooka K (2005). Functional analysis of two solanesyl diphosphate synthases from *Arabidopsis thaliana*. Biosci Biotechnol Biochem..

[evu191-B37] Jiao Y (2011). Ancestral polyploidy in seed plants and angiosperms. Nature.

[evu191-B38] Jun L, Saiki R, Tatsumi K, Nakagawa T, Kawamukai M (2004). Identification and subcellular localization of two solanesyl diphosphate synthases from *Arabidopsis thaliana*. Plant Cell Physiol..

[evu191-B39] Kassahn KS, Dang VT, Wilkins SJ, Perkins AC, Ragan MA (2009). Evolution of gene function and regulatory control after whole-genome duplication: comparative analyses in vertebrates. Genome Res..

[evu191-B40] Kawashima CG, Berkowitz O, Hell R, Noji M, Saito K (2005). Characterization and expression analysis of a serine acetyltransferase gene family involved in a key step of the sulfur assimilation pathway in *Arabidopsis*. Plant Physiol..

[evu191-B41] Kim SH, Yi SV (2006). Correlated asymmetry of sequence and functional divergence between duplicate proteins of *Saccharomyces cerevisiae*. Mol Biol Evol..

[evu191-B42] Kishino H, Hasegawa M (1989). Evaluation of the maximum likelihood estimate of the evolutionary tree topologies from DNA sequence data, and the branching order in hominoidea. J Mol Evol..

[evu191-B43] Kosugi S, Ohashi Y (2002). E2Ls, E2F-like repressors of *Arabidopsis* that bind to *E2F* sites in a monomeric form. J Biol Chem..

[evu191-B44] Kunieda T (2013). Spatiotemporal secretion of *PEROXIDASE36* is required for seed coat mucilage extrusion in *Arabidopsis*. Plant Cell.

[evu191-B45] Kwon C (2008). Co-option of a default secretory pathway for plant immune responses. Nature.

[evu191-B46] Lan P, Li W, Wang H, Ma W (2010). Characterization, sub-cellular localization and expression profiling of the isoprenylcysteine methylesterase gene family in *Arabidopsis thaliana*. BMC Plant Biol..

[evu191-B47] Liu SL, Adams KL (2008). Molecular adaptation and expression evolution following duplication of genes for organellar ribosomal protein S13 in rosids. BMC Evol Biol..

[evu191-B48] Liu SL, Adams KL (2010). Dramatic change in function and expression pattern of a gene duplicated by polyploidy created a paternal effect gene in the Brassicaceae. Mol Biol Evol..

[evu191-B49] Liu SL, Baute GJ, Adams KL (2011). Organ and cell type-specific complementary expression patterns and regulatory neofunctionalization between duplicated genes in *Arabidopsis thaliana*. Genome Biol Evol..

[evu191-B50] Lu SX, Hrabak EM (2002). An *Arabidopsis* calcium-dependent protein kinase is associated with the endoplasmic reticulum. Plant Physiol..

[evu191-B51] Lynch M, Force A (2000). The probability of duplicate gene preservation by subfunctionalization. Genetics.

[evu191-B52] Mahfouz MM, Kim S, Delauney AJ, Verma DP (2006). *Arabidopsis* TARGET OF RAPAMYCIN interacts with RAPTOR, which regulates the activity of S6 kinase in response to osmotic stress signals. Plant Cell.

[evu191-B53] Marques AC, Vinckenbosch N, Brawand D, Kaessmann H (2008). Functional diversification of duplicate genes through subcellular adaptation of encoded proteins. Genome Biol..

[evu191-B54] Matre P, Meyer C, Lillo C (2009). Diversity in subcellular targeting of the *PP2A B'eta* subfamily members. Planta.

[evu191-B55] Millar AH, Whelan J, Small I (2006). Recent surprises in protein targeting to mitochondria and plastids. Curr Opin Plant Biol..

[evu191-B56] Mollier P, Hoffmann B, Debast C, Small I (2002). The gene encoding *Arabidopsis thaliana* mitochondrial ribosomal protein S13 is a recent duplication of the gene encoding plastid S13. Curr Genet..

[evu191-B57] Murcha MW (2007). Characterization of the preprotein and amino acid transporter gene family in *Arabidopsis*. Plant Physiol..

[evu191-B58] Neuberger G, Maurer-Stroh S, Eisenhaber B, Hartig A, Eisenhaber F (2003). Prediction of peroxisomal targeting signal 1 containing proteins from amino acid sequence. J Mol Biol..

[evu191-B59] Noji M, Inoue K, Kimura N, Gouda A, Saito K (1998). Isoform-dependent differences in feedback regulation and subcellular localization of serine acetyltransferase involved in cysteine biosynthesis from *Arabidopsis thaliana*. J Biol Chem..

[evu191-B60] Okada K, Saito T, Nakagawa T, Kawamukai M, Kamiya Y (2000). Five geranylgeranyl diphosphate synthases expressed in different organs are localized into three subcellular compartments in *Arabidopsis*. Plant Physiol..

[evu191-B61] Ostersetzer O, Kato Y, Adam Z, Sakamoto W (2007). Multiple intracellular locations of Lon protease in *Arabidopsis*: evidence for the localization of *AtLon4* to chloroplasts. Plant Cell Physiol..

[evu191-B62] Owens SM, Harberson NA, Moore RC (2013). Asymmetric functional divergence of young, dispersed gene duplicates in *Arabidopsis thaliana*. J Mol Evol..

[evu191-B63] Panchin AY, Gelfand MS, Ramensky VE, Artamonova (2010). Asymmetric and non-uniform evolution of recently duplicated human genes. Biol Direct..

[evu191-B64] Pond SLK, Frost SDW, Muse SV (2004). HyPhy: hypothesis testing using phylogenies. Bioinformatics.

[evu191-B65] Proost S (2009). PLAZA: a comparative genomics resource to study gene and genome evolution in plants. Plant Cell.

[evu191-B66] Reumann S (2004). Specification of the peroxisome targeting signals type 1 and type 2 of plant peroxisomes by bioinformatics analyses. Plant Physiol..

[evu191-B67] Reumann S (2007). Proteome analysis of *Arabidopsis* leaf peroxisomes reveals novel targeting peptides, metabolic pathways, and defense mechanisms. Plant Cell.

[evu191-B68] Sanderfoot A (2007). Increases in the number of SNARE genes parallels the rise of multicellularity among the green plants. Plant Physiol..

[evu191-B69] Sanmartín M (2007). Divergent functions of *VTI12* and *VTI11* in trafficking to storage and lytic vacuoles in *Arabidopsis*. Proc Natl Acad Sci U S A..

[evu191-B70] Schmidt HA, Strimmer K, Vingron M, von Haeseler A (2002). TREE-PUZZLE: maximum likelihood phylogenetic analysis using quartets and parallel computing. Bioinformatics.

[evu191-B71] Schmid M (2005). A gene expression map of *Arabidopsis thaliana* development. Nat Genet..

[evu191-B72] Schnable JC, Wang X, Pires JC, Freeling M (2012). Escape from preferential retention following repeated whole genome duplications in plants. Front Plant Sci..

[evu191-B73] Schultz CJ, Coruzzi GM (1995). The aspartate aminotransferase gene family of *Arabidopsis* encodes isoenzymes localized to three distinct subcellular compartments. Plant J..

[evu191-B75] Shimodaira H, Hasegawa M (2001). CONSEL: for assessing the confidence of phylogenetic tree selection. Bioinformatics.

[evu191-B76] Soltis DE, Soltis PE, Endress PK, Chase MW (2005). Phylogeny and evolution of angiosperms.

[evu191-B77] Stamatakis A (2006). RAxML-VI-HPC: maximum likelihood-based phylogenetic analyses with thousands of taxa and mixed models. Bioinformatics.

[evu191-B78] Storey JD, Tibshirani R (2003). Statistical significance for genomewide studies. Proc Natl Acad Sci U S A..

[evu191-B79] Surpin M, Raikhel N (2004). Traffic jams affect plant development and signal transduction. Nat Rev Mol Cell Biol..

[evu191-B80] Székely G (2008). Duplicated P5CS genes of *Arabidopsis* play distinct roles in stress regulation and developmental control of proline biosynthesis. Plant J..

[evu191-B81] Szklarczyk R, Huynen MA (2009). Expansion of the human mitochondrial proteome by intra- and inter-compartmental protein duplication. Genome Biol..

[evu191-B82] Teng YS, Chan PT, Li HM (2012). Differential age-dependent import regulation by signal peptides. PLoS Biol..

[evu191-B83] Uemura T (2004). Systematic analysis of SNARE molecules in *Arabidopsis*: dissection of the post-Golgi network in plant cells. Cell Struct Funct..

[evu191-B85] Voelker C, Schmidt D, Mueller-Roeber B, Czempinski K (2006). Members of the *Arabidopsis AtTPK*/KCO family form homomeric vacuolar channels in planta. Plant J..

[evu191-B86] Wang X, Huang Y, Lavrov DV, Gu X (2009). Comparative study of human mitochondrial proteome reveals extensive protein subcellular relocalization after gene duplications. BMC Evol Biol..

[evu191-B87] Williams P, Hardeman K, Fowler J, Rivin C (2006). Divergence of duplicated genes in maize: evolution of contrasting targeting information for enzymes in the porphyrin pathway. Plant J..

[evu191-B88] Yang Z (2007). PAML 4: phylogenetic analysis by maximum likelihood. Mol Biol Evol..

[evu191-B89] Zhang L (2011). *Arabidopsis* R-SNARE proteins *VAMP721* and *VAMP722* are required for cell plate formation. PLoS One.

