# Supplementary material for: Protein Subcellular Relocalization of Duplicated Genes in Arabidopsis
Source: Genome Biol Evol. 2014 Sep 4;6(9):2501–15. doi: 10.1093/gbe/evu191 (PMC4202327; doi:10.1093/gbe/evu191)
Supplement: Supplementary Data [file supp_6_9_2501__index.html]

Protein subcellular relocalization of duplicated genes in Arabidopsis — Protein Subcellular Relocalization of Duplicated Genes in Arabidopsis — Supplementary Data 

# Protein Subcellular Relocalization of Duplicated Genes in *Arabidopsis*

## Supplementary Data

files

**Files in this Data Supplement:**

- Supplementary Data - pdf file
- Supplementary Data - pdf file
- Supplementary Data - xls file
- Supplementary Data - xls file
