# Supplementary material for: Protein Subcellular Relocalization of Duplicated Genes in Arabidopsis
Source: Genome Biol Evol. 2014 Sep 4;6(9):2501–15. doi: 10.1093/gbe/evu191 (PMC4202327; doi:10.1093/gbe/evu191)
Supplement: Supplementary Data [file supp_evu191_Supplemental_figures.pdf]

Supplemental figure S1

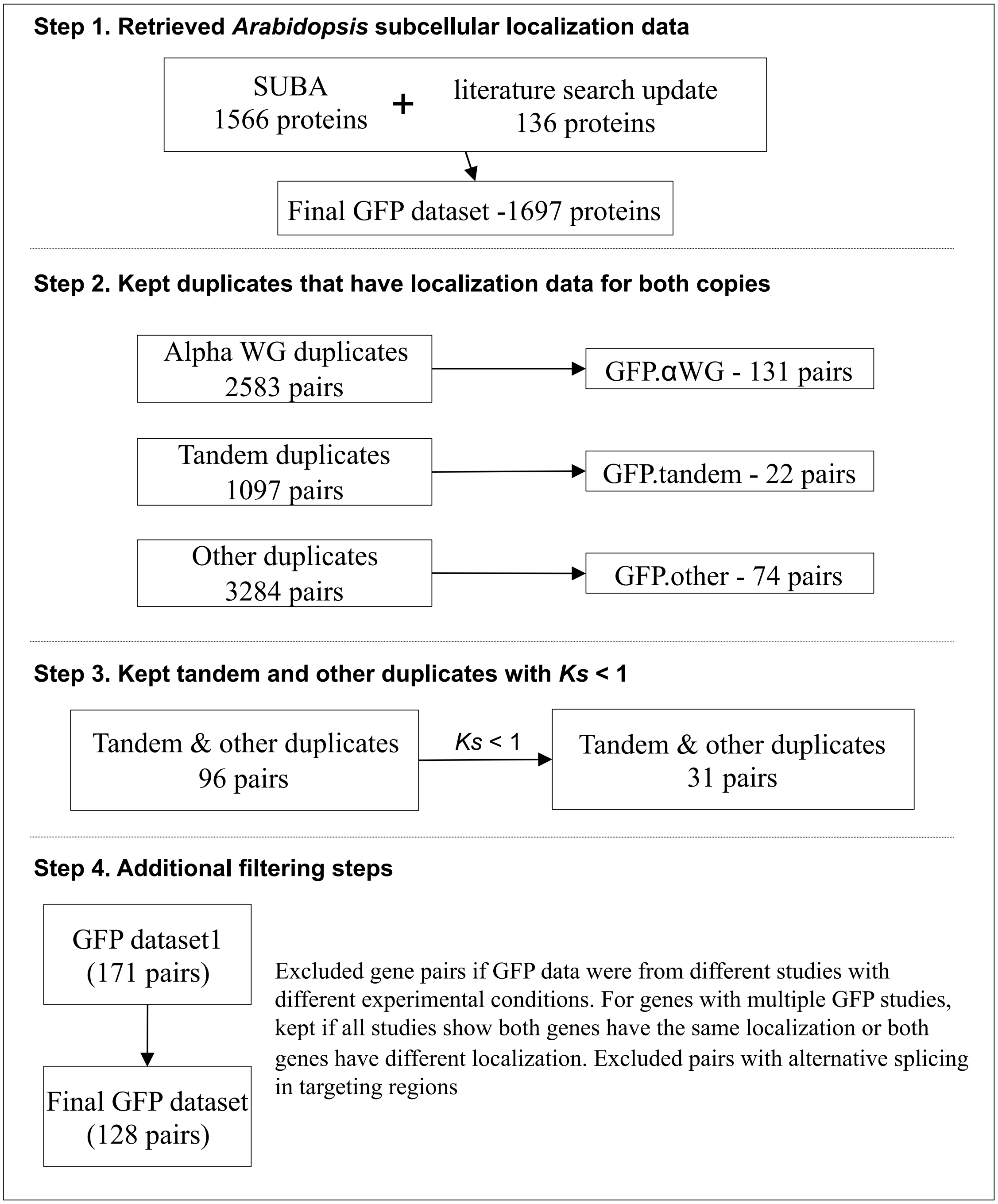

A flowchart of duplicate gene dataset construction and all filtration steps.

Supplemental figure S2

A

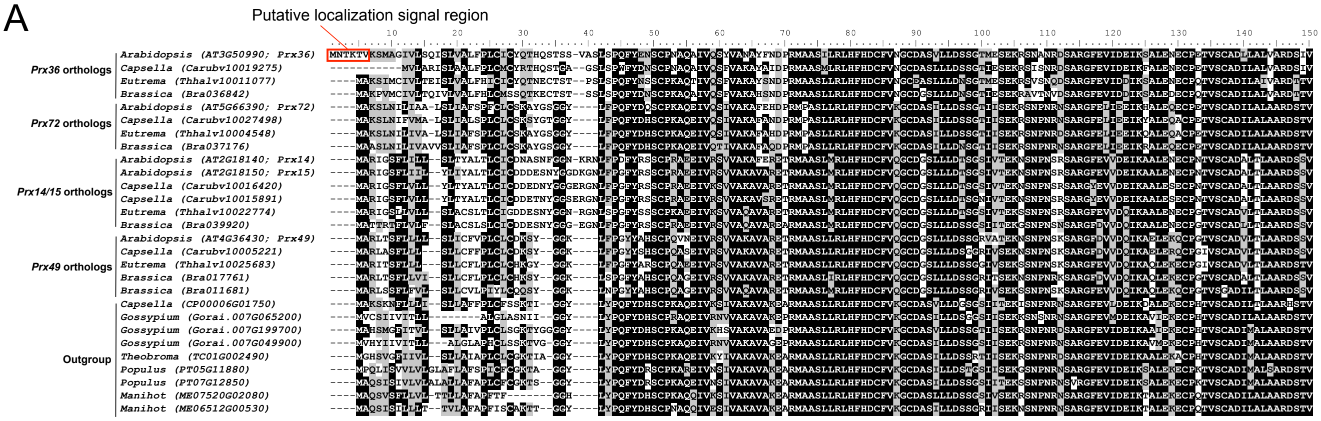

B

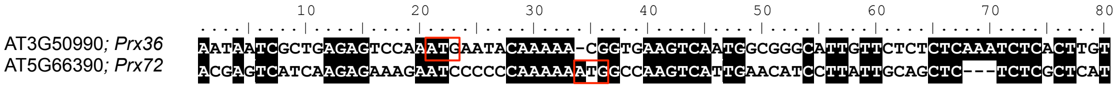

Amino acid and genomic sequence alignment for PRX36, PRX72, and their orthologs. (A) Amino acid sequence alignment of the N-terminus of PRX36 (AT3G50990), *PRX72* (AT5G66390), and their orthologs in the outgroup species showing the putative targeting signal peptide in PRX36. The *Vitis* ortholog was excluded due to its incomplete sequence. (B) Genomic sequence alignment between *Prx36* and *Prx72* showing the abolishment of the original start codon and the acquisition of a new start codon in *Prx36*.

Supplementary figure S3

A) Microarray gene expression among paralogs of *Prx36*

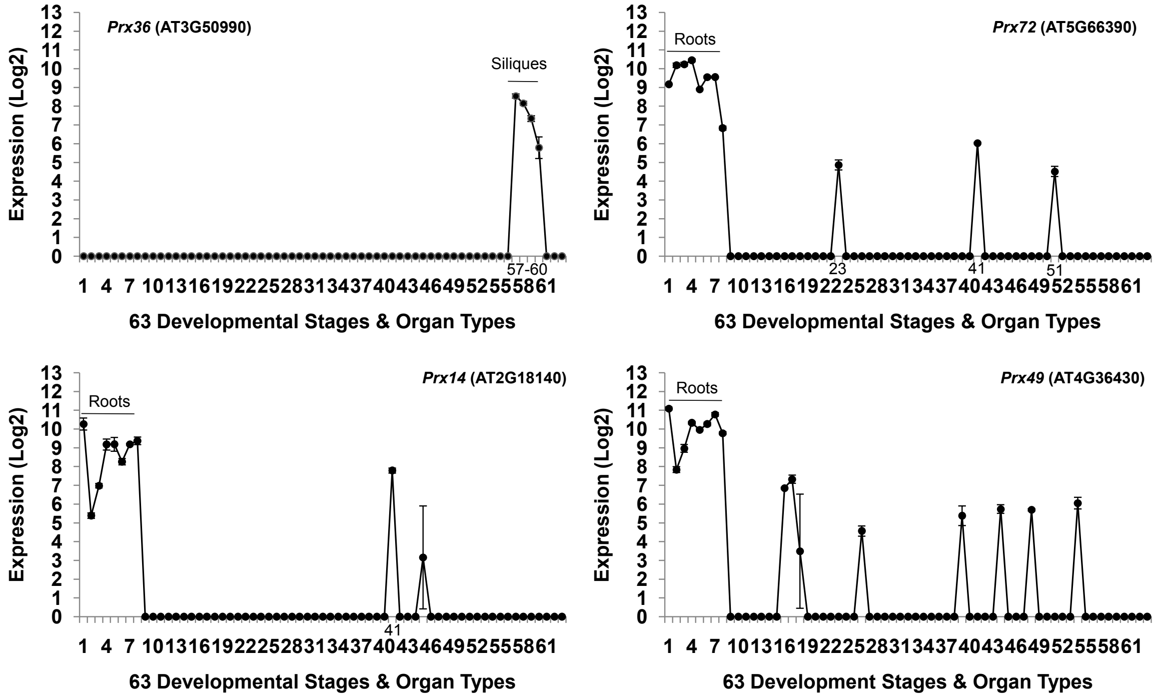

B) Microarray gene expression between *CPK2* and *CPK1*

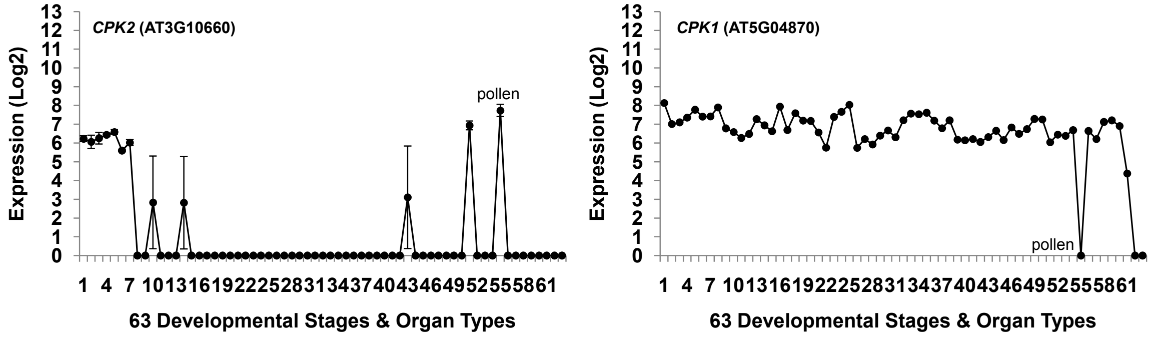

MAS5-normalized microarray gene expression data from 63 different developmental stages and organ types in *Arabidopsis thaliana*. Error bars indicate standard deviations (n = 3). The 63 different developmental stages and organ types are listed in Table S4. (A) Microarray gene expression among paralogs of *Prx36* showing silique-specific expression pattern in *Prx36* and mostly root-specific expression pattern in other paralogs. (B) Microarray gene expression between *CPK2* and *CPK1* showing a complementary expression profile in which *CPK2* is expressed in pollen and *CPK1* is not expressed in pollen.

Methods: Raw Affymetrix ATH1 microarray data from 63 different organ types and developmental stages (Schmid et al. 2005) were obtained from the TAIR website (http://www.arabidopsis.org/). Raw ATH1 microarray files in a CEL format were processed and normalized using the MAS5.0 algorithm in Bioconductor (http://www.bioconductor.org/). To determine the absence or presence of gene expression, the “mas5calls” function in Bioconductor was used with the Wilcoxon signed rank-based gene expression absence/presence detection algorithm. This statistical test determined if the expression signal was significantly greater than background noise and yielded a probability value. For each microarray chip, genes with a probability value less than 0.05 were considered as presence of expression, whereas genes with a probability value equal to or greater than 0.05 were considered as absence of expression. Since there are three different biological replicates, genes that showed expression under at least two different biological replicates were assigned as presence of expression. The determination of absence and presence of gene expression was only applied for the estimation of asymmetric expression index, but not for the estimation of tissue specificity index.

Supplementary figure S4

A) Topology test

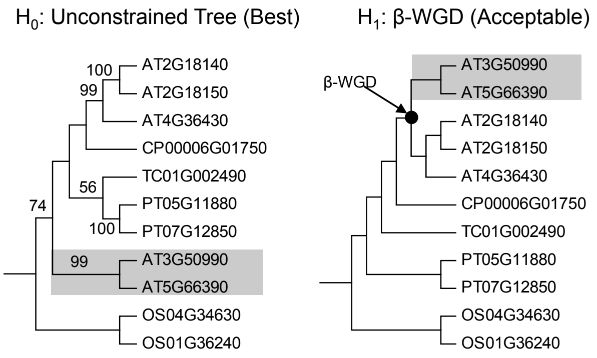

COMPARISON OF USER TREES (NO CLOCK)

Tree	log L	difference	S.E.	p-1sKH	p-SH	c-ELW	2sKH
H_0	-8126.50	0.00 <---- best		1.0000 +	1.0000 +	0.8888 +	best
H_1	-8140.02	13.53	11.1020	0.0960 +	0.1200 +	0.1112 +	+

Abbreviation: 1sKH , one sided KH test based on pairwise SH tests; SH, Shimodaira-Hasegawa test; ELW, Expected Likelihood Weight; 2sKH, two sided Kishino-Hasegawa test.

Plus signs denote the confidence sets. Minus signs denote significant exclusion. All tests used 5% significance level. 1sKH, SH, and ELW performed 1000 resamplings using the RELL method. 1sKH and 2sKH are correct to the 2nd position after the decimal point of the log-likelihoods.

B) *dN* tree

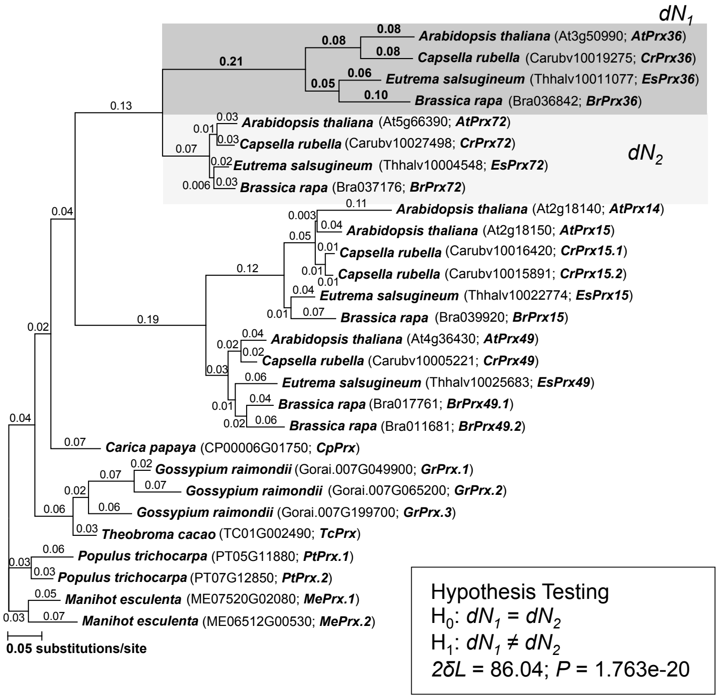

Figure S4. Topology test and sequence sequence rate analyses of *Prx36* and *Prx72*. (A) Topology test between the unconstrained tree inferred from a maximum likelihood phylogenetic analysis (i.e., the best tree) and the alternative tree that assumes the species tree relationships, using the software TREE-PUZZLE. Three different tests (KH test, Kishino-Hasegawa test; SH test, Shimodaira-Hasegawa test; and ELW test, Expected Likelihood Weight test) were used for the topology tests. The constrained species tree topology was used for subsequent rate analyses if the constrained tree is not statistically rejected. The box summarizes the statistics of different tree topology tests. The 500 bootstrapping replicates of maximum likelihood analyses are shown on the branches in the unconstrained tree. The monocot, *Oryza sativa*, was used as the outgroup. The *Vitis* ortholog was excluded due to its incomplete sequence.
(B) The *dN* tree inferred using the software HyPhyl showing that the *dN* of *Prx36* evolved significantly faster than that of *Prx72*. Species include: At – *Arabidopsis thaliana*, Al – *Arabidopsis lyrata*, Cr – *Capsella rubella*, Es – *Eutrema salsugineum*, Br – *Brassica rapa*, Cp – *Carica papaya*, Gr – *Gossypium raimondii*, Tc – *Theobroma cacao*, Pt – *Populus trichocarpa*, and Me – *Manihot esculenta.*

Supplementary figure S5

A) Topology test

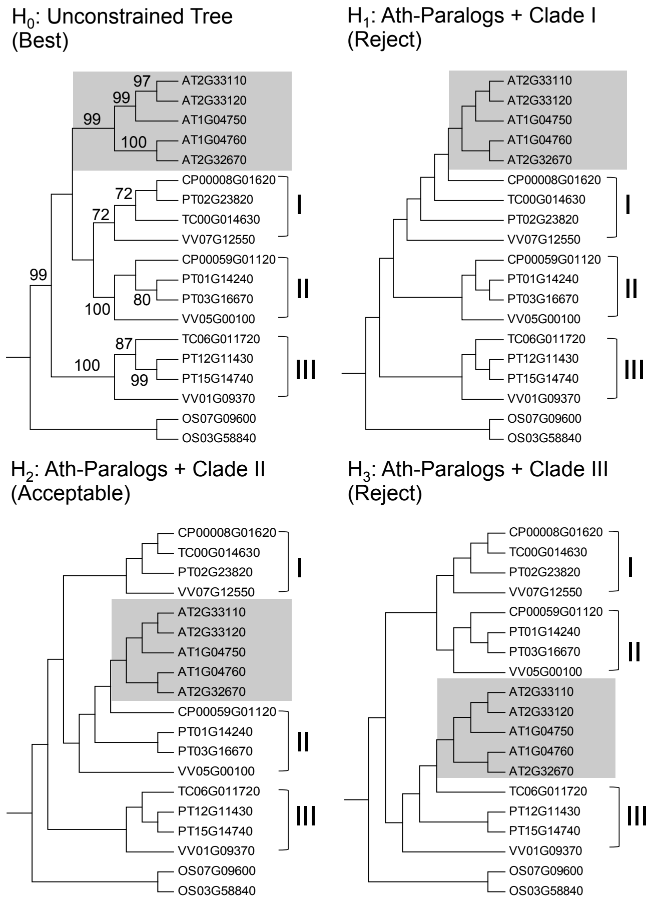

COMPARISON OF USER TREES (NO CLOCK)

Tree	log L	difference	S.E.	p-1sKH	p-SH	c-ELW	2sKH
H_0	-6238.96	0.00	<---- best	1.0000 +	1.0000 +	0.7234 +	best
H_1	-6271.25	32.28	12.4081	0.0080 -	0.0720 +	0.0013 -	-
H_2	-6246.67	7.71	12.6437	0.2700 +	0.5700 +	0.2753 +	+
H_3	-6329.93	90.97	20.0548	0.0000 -	0.0000 -	0.0000 -	-

Abbreviation: 1sKH , one sided KH test based on pairwise SH tests; SH, Shimodaira-Hasegawa test; ELW, Expected Likelihood Weight; 2sKH, two sided Kishino-Hasegawa test.

Plus signs denote the confidence sets. Minus signs denote significant exclusion. All tests used 5% significance level. 1sKH, SH, and ELW performed 1000 resamplings using the RELL method. 1sKH and 2sKH are correct to the 2nd position after the decimal point of the log-likelihoods.

B) *dN* tree

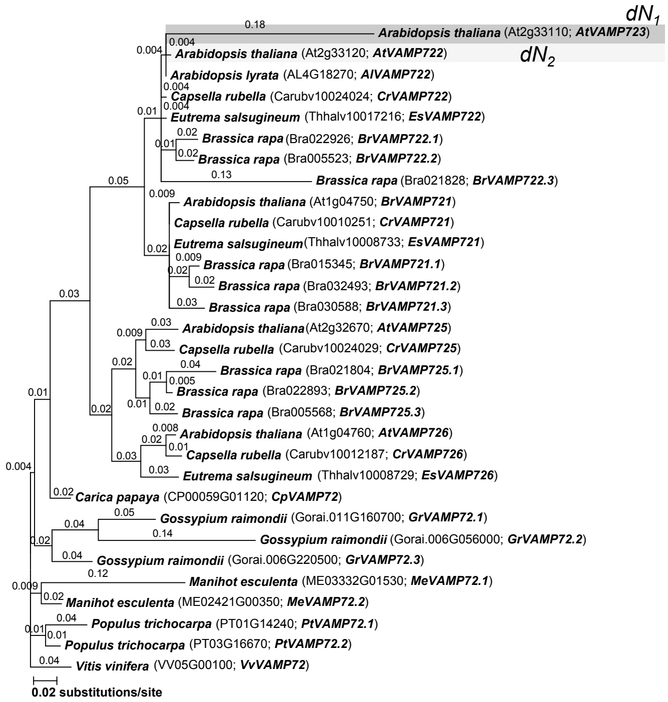

Hypothesis Testing
H_0: $dN_1 = dN_2$
H_1: $dN_1 \neq dN_2$
$2\delta L = 48.11$; $P = 4.030e-12$

C) Gene expression assay by RT-PCR

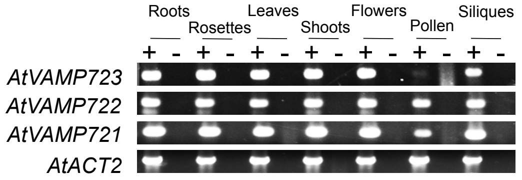

Figure S5. Topology test, sequence rate analyses, and gene expression assays of *VAMP723*, *VAMP722*, and their *Arabidopsis* paralog (*VAMP721*). (A) Topology test between the unconstrained tree inferred from a maximum-likelihood phylogenetic analysis (i.e., the best tree) and the alternative tree that assumes the species tree relationships, using the software TREE-PUZZLE. See figure S4 legend for methods details.

(B) The *dN* tree inferred using the software HyPhyl showing that the *dN* of *VAMP723* evolved significantly faster than that of *VAMP722*. Species include: At – *Arabidopsis thaliana*, Al – *Arabidopsis lyrata*, Cr – *Capsella rubella*, Es – *Eutrema salsugineum*, Br – *Brassica rapa*, Cp – *Carica papaya*, Gr – *Gossypium raimondii*, Tc – *Theobroma cacao*, Pt – *Populus trichocarpa*, Me – *Manihot esculenta*, and Vv – *Vitis vinifera.*

(C) Reverse transcription (RT)-PCR expression assays of *VAMP723*, *VAMP722*, and *VAMP721*. Plus signs (+) indicate reactions with reverse transcriptase and minus signs (-) indicate reactions without reverse transcriptase. *ACT2* was a positive control.

Supplementary figure S6

A) Topology Test

H_0: Unconstrained Tree (Best) H_1: Tandem Duplication (Acceptable)

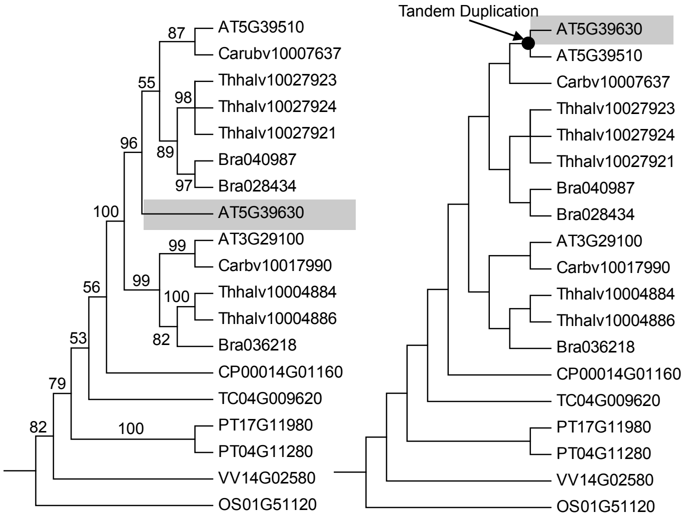

COMPARISON OF USER TREES (NO CLOCK)

Tree	log L	difference	S.E.	p-1sKH	p-SH	c-ELW	2sKH
H0	-5889.37	0.00	<---- best	1.0000 +	1.0000 +	0.9461 +	best
H1	-5896.01	6.65	3.9575	0.0470 -	0.0540 +	0.0539 +	+

Abbreviation: 1sKH , one sided KH test based on pairwise SH tests; SH, Shimodaira-Hasegawa test; ELW, Expected Likelihood Weight; 2sKH, two sided Kishino-Hasegawa test.

Plus signs denote the confidence sets. Minus signs denote significant exclusion. All tests used 5% significance level. 1sKH, SH, and ELW performed 1000 resamplings using the RELL method. 1sKH and 2sKH are correct to the 2nd position after the decimal point of the log-likelihoods.

B) α-WGD Syntenic Block

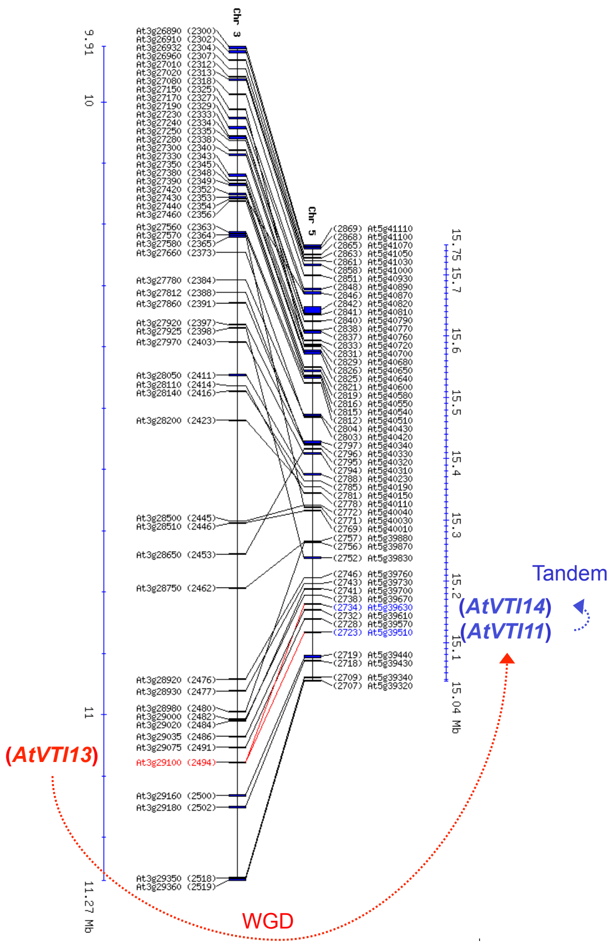

C) *dN* tree

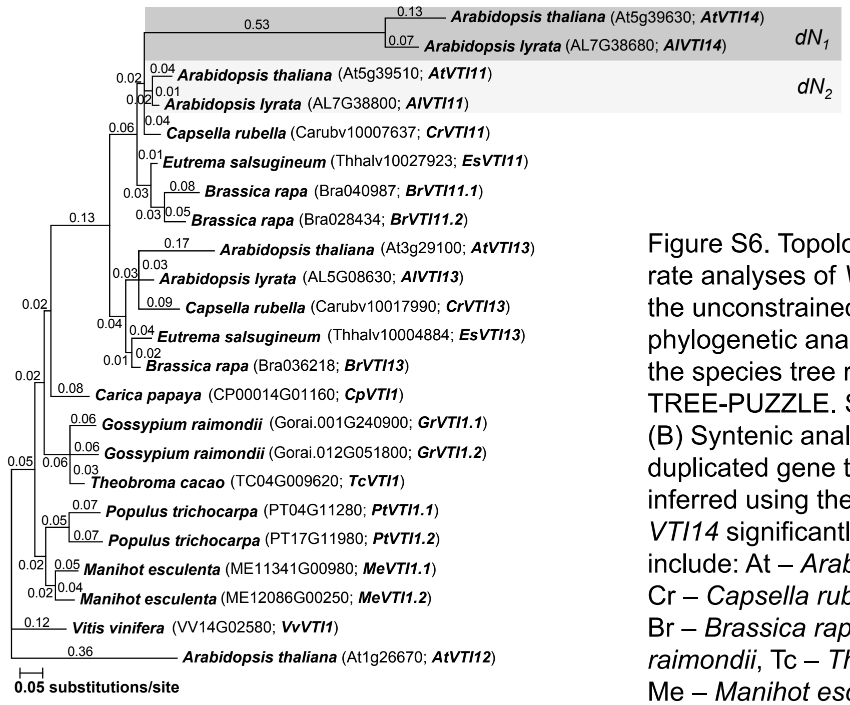

Hypothesis Testing
H_0: $dN_1 = dN_2$
H_1: $dN_1 \neq dN_2$
$2\delta L$ = 121.18; P = 3.490e-28

Figure S6. Topology test, syntenic analysis, and sequence rate analyses of *VTI14* and *VTI11*. (A) Topology test between the unconstrained tree inferred from a maximum-likelihood phylogenetic analysis and the alternative tree that assumes the species tree relationships, using the software TREE-PUZZLE. See figure S4 legend for methods details. (B) Syntenic analysis showing that *VTI14* is a tandemly duplicated gene that originated from *VTI11*. (C) The *dN* tree inferred using the software HyPhyl showing that the *dN* of *VTI14* significantly evolved faster than that of *VTI11*. Species include: At – *Arabidopsis thaliana*, Al – *Arabidopsis lyrata*, Cr – *Capsella rubella*, Es – *Eutrema salsugineum*, Br – *Brassica rapa*, Cp – *Carica papaya*, Gr – *Gossypium raimondii*, Tc – *Theobroma cacao*, Pt – *Populus trichocarpa*, Me – *Manihot esculenta*, and Vv – *Vitis vinifera*.

Supplementary figure S7

A) Topology test

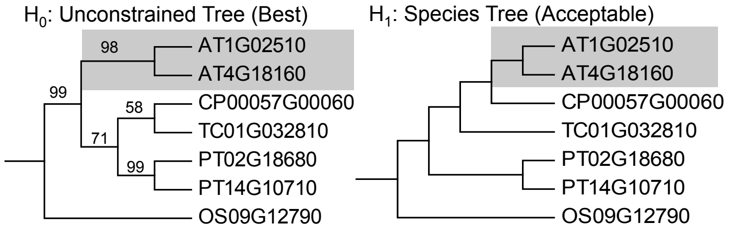

COMPARISON OF USER TREES (NO CLOCK)

Tree	log L	difference	S.E.	p-1sKH	p-SH	c-ELW	2sKH
H_0	-7950.04	0.00	<---- best	1.0000 +	1.0000 +	0.9094 +	best
H_1	-7960.22	10.18	7.8011	0.0950 +	0.0880 +	0.0906 +	+

Abbreviation: 1sKH , one sided KH test based on pairwise SH tests; SH, Shimodaira-Hasegawa test; ELW, Expected Likelihood Weight; 2sKH, two sided Kishino-Hasegawa test.

Plus signs denote the confidence sets. Minus signs denote significant exclusion. All tests used 5% significance level. 1sKH, SH, and ELW performed 1000 resamplings using the RELL method. 1sKH and 2sKH are correct to the 2nd position after the decimal point of the log-likelihoods.

B) *dN* tree

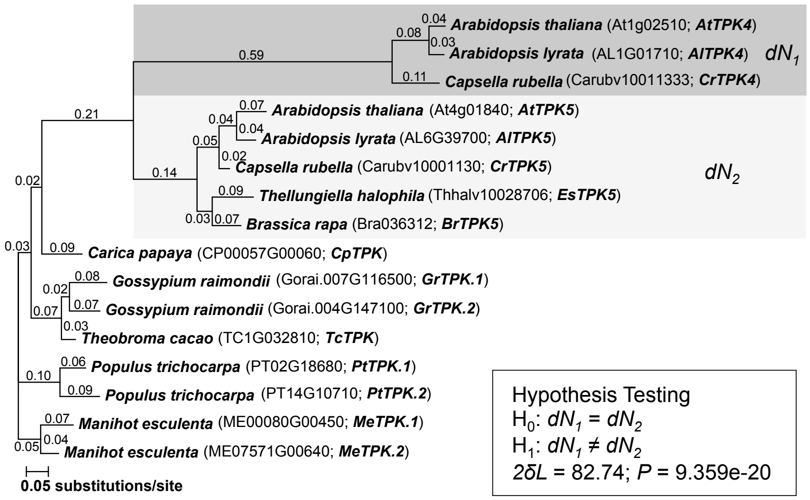

Figure S7. Topology test and sequence rate evolution of *TPK4* and *TPK5*. The *Vitis* ortholog was not used in the analysis due to its incomplete sequence. (A) Topology test, using the software TREE-PUZZLE, between the unconstrained tree inferred from a maximum likelihood phylogenetic analysis and the alternative tree topology, assuming the species tree relationship, showing that the species tree relationship is not rejected. See figure S4 legend for methods details.
(B) The *dN* tree inferred using the software HyPhyl showing that *TPK4* evolved significantly faster than *TPK5*. Species include: At – *Arabidopsis thaliana*, Al – *Arabidopsis lyrata*, Cr – *Capsella rubella*, Es – *Eutrema salsugineum*, Br – *Brassica rapa*, Cp – *Carica papaya*, Gr – *Gossypium raimondii*, Tc – *Theobroma cacao*, Pt – *Populus trichocarpa*, and Me – *Manihot esculenta.*

Supplementary figure S8

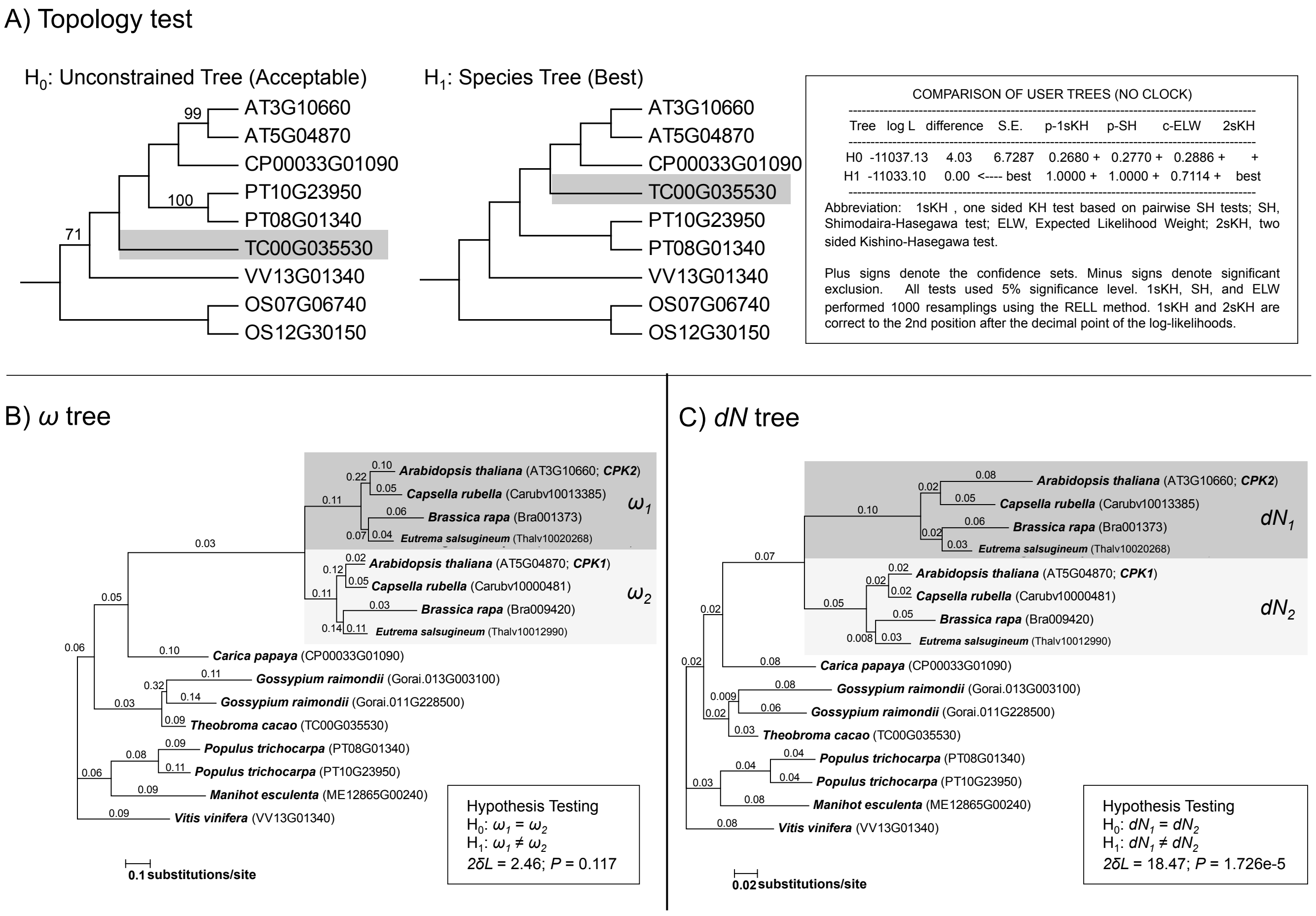

Figure S8. Topology test and sequence rate evolution in *CPK1* and *CPK2*. (A) Topology test, using the software TREE-PUZZLE, between the unconstrained tree inferred from a maximum likelihood phylogenetic analysis and the alternative tree assuming the species tree relationship showing that the species tree topology is not significantly rejected. See figure S4 legend for methods details. (B) The *dN*/*dS* ratio (= ω) tree inferred using the software PAML showing no significant ω difference between *CPK1* and *CPK2*. (C) The *dN* tree inferred using the software HyPhyl showing that *CPK2* has significantly higher *dN* values than *CPK1*. Species include in (B) and (C): At – *Arabidopsis thaliana*, Al – *Arabidopsis lyrata*, Cr – *Capsella rubella*, Es – *Eutrema salsugineum*, Br – *Brassica rapa*, Cp – *Carica papaya*, Gr – *Gossypium raimondii*, Tc – *Theobroma cacao*, Pt – *Populus trichocarpa*, Me – *Manihot esculenta*, and Vv – *Vitis vinifera.*

Supplementary figure S9

A

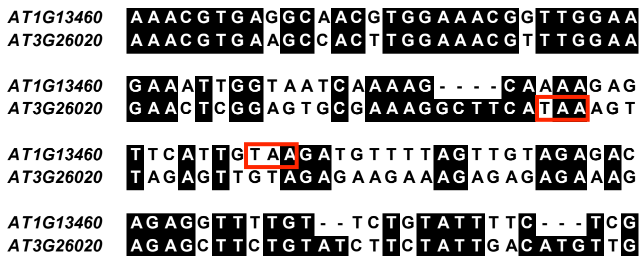

B) PTS1 prediction for peroxisome targeting

	Taxa/Gene Name	Score	C-terminus	Prediction
PP2A B' θ **orthologs**	***Arabidopsis*** **(AT1G13460; *PP2A B' θ*)**	-5.909	EEIGNQKQKSSL	Targeted
	Capsella **(Carubv10008945)**	-5.909	EEIGNQKQKSSL	Targeted
	Eutrema **(Thhalv10007450)**	-40.301	EEIGMQKQKSSS	Not targeted
	Brassica **(Bra019720)**	-59.973	EIGMQKQKSSSS	Not targeted
PP2A B' η **orthologs**	***Arabidopsis*** **(AT3G26020; *PP2A B 'η*)**	-29.670	KRLEELGVRKAS	Not targeted
	Capsella **(Carubv10019373)**	-32.109	KRLEELGVQKAS	Not targeted
	Eutrema **(Thhalv10004020)**	-29.670	KRLEELGVRKAS	Not targeted
	Brassica **(Bra025178)**	-37.327	RRLEEVGMRKAS	Not targeted
**Outgroup**	***Theobroma*** **(TC02G026330)**	-71.508	VSHKANANGSSG	Not targeted
	Gossypium **(Gorai.012G174400)**	-60.528	ETSESMANGSSG	Not targeted
	Populus **(PT10G05310)**	-58.004	KTASDDTVVVPC	Not targeted
	Populus **(PT08G17820)**	-55.895	APRKALTCSASG	Not targeted
	Manihot **(ME04175G00800)**	-78.870	LATQKASSNKAS	Not targeted
	Vitis **(VV01G03580)**	-71.521	KRVASNEAVLVP	Not targeted

Genomic sequence alignment and subcellular targeting prediction of *PP2A B'θ*, *PP2A B'η*, and their orthologs. (A) Genomic sequence alignment between *PP2A B' θ* and *PP2A B' η* in *Arabidopsis thaliana* showing that the ancestral stop codon in *PP2A B' θ* probably was abolished by a point mutation and gained new stop codon by two point mutations in the 3' downstream region. (B) Subcellular targeting prediction inferred using the program PTS1 showing that *PP2A B' θ* was relocalized to peroxisomes in *Arabidopsis* and *Capsella* via the gain of the peroxisomal targeting signal peptides (underline).

Supplementary figure S10

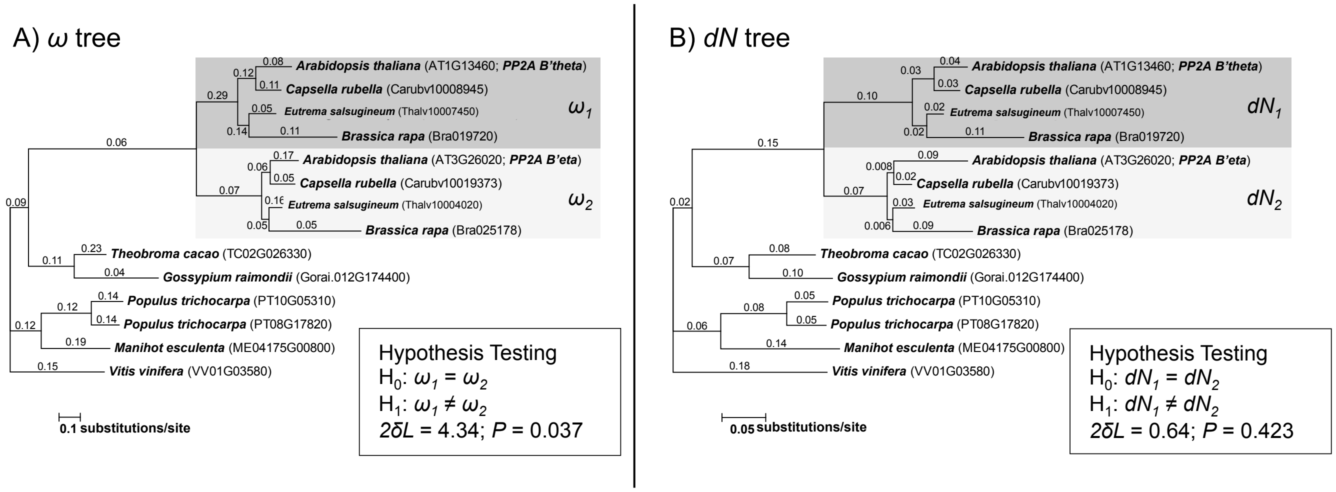

Sequence rate analyses of *PP2A B' θ* and *PP2A B' η*. The *Carica* ortholog was not used in the analysis due to its incomplete sequence. (A) The *dN*/*dS* ratio (= *ω*) tree inferred using the software PAML showing that *PP2A B' θ* has a significantly higher *ω* value than *PP2A B' η*. (B) The *dN* tree inferred using the software HyPhyl showing that *PP2A B' θ* and *PP2A B' η* did not evolve in a symmetric fashion. Species include: At – *Arabidopsis thaliana*, Al – *Arabidopsis lyrata*, Cr – *Capsella rubella*, Es – *Eutrema salsugineum*, Br – *Brassica rapa*, Cp – *Carica papaya*, Gr – *Gossypium raimondii*, Tc – *Theobroma cacao*, Pt – *Populus trichocarpa*, Me – *Manihot esculenta*, and Vv – *Vitis vinifera*.
