# Supplementary material for: Protein Subcellular Relocalization of Duplicated Genes in Arabidopsis
Source: Genome Biol Evol. 2014 Sep 4;6(9):2501–15. doi: 10.1093/gbe/evu191 (PMC4202327; doi:10.1093/gbe/evu191)
Supplement: Supplementary Data [file supp_evu191_Tables_S1_and_S2.pdf]

Table S1. List of gene-specific primers used for the RT-PCR assays.

| Species | Gene_ID | Direction | Primer (5'->3') | Locus_ID/GenBank_ID |
|---|---|---|---|---|
| *Carica papaya* | *CpACT1* | Forward | AGACACACAGGTGTCATGGTTGGA | EL784289 |
| | | Reverse | GGCAGTTTCAAGCTCCTGCTCAAA | |
| | *CpCPK* | Forward | ACAGGTCCTGCATGGTGATCTTGA | CP00033G01090 |
| | | Reverse | TGCAACGTAGCAGCAACAAACTCC | |
| | *CpTPK* | Forward | AAGACCACCCACAACAACGATTGC | CP00057G00060 |
| | | Reverse | CCAACAAGCACGAAGACACAAGCA | |
| | *CpVTI* | Forward | ACTGCGAGCTCTCCGCTAATCTTT | CP00014G01160 |
| | | Reverse | TTTGATCGGCTGATGCCGTCATTG | |
| *Gossypium hirsutum* | *GhACT1* | Forward | ACTGGTGTTATGGTTGGGATGGGT | ES812773 |
| | | Reverse | AGCTTGGATGGCAACATACATGGC | |
| | *GhCPK* | Forward.uni | GGATGCAGTGGCAGTTCATGTTGT | ES795182, ES817674, DW477354, ES829211 |
| | | Reverse.uni | TTGAGAAGTGCATCCTCCTGCTGA | |
| | *GhTPK.1* | Forward | ACCCGGGTGTTGATGCTCTTTACT | ES828970 |
| | | Reverse | AAGGTTCCAATTCCAACGCACAGC | |
| | *GhTPK.2* | Forward | ACCCGGTTGTTGATGCTCTCTACT | ES811988 |
| | | Reverse | AATCAACCCAGTCCAAGCTCTCCA | |
| | *GhVTI.1* | Forward | GAATATTGTCGAAAGCCACCGAGC | DR455009 |
| | | Reverse | GCTTCTGCATCTTCTAGTCTGGCT | |
| | *GhVTI.2* | Forward | AAAGCCACCACTGAGGATCG | ES824385 |
| | | Reverse | TCTGCATCTTCCACTCCAGCTCTT | |
| *Arabidopsis thaliana* | *AtPrx14* | Forward | TCGTTCAGGGTTGTGATGGATCGT | AT2G18140 |
| | | Reverse | CAAGTTGTCGGGTTCAGGAAGGTCT | |
| | *AtPrx15* | Forward | TCGTTCAGGGTTGTGATGGATCGT | AT2G18150 |
| | | Reverse | AGTGTTGTTGGGTGCAGGAATGTTG | |
| | *AtPrx36* | Forward | GGCGGGCATTGTTCTCTCTCAAAT | AT3G50990 |
| | | Reverse | TCAATGACTTCGAACCCTCGAGCA | |
| | *AtPrx72* | Forward | GAAACCACGGGCTGAGTTTCTGTT | AT5G66390 |
| | | Reverse | GCAGCTCTCTCGCTCATTGCATTT | |
| | *AtVAMP721* | Forward | AGACGGTCGATTGGATCGGAGATT | AT1G04750 |
| | | Reverse | TGAAGGTATGACCGTCGCAGTTGT | |
| | *AtVAMP722* | Forward | CTTCAACTACGTTAAGATTTCTCCTACAC | AT2G33120 |
| | | Reverse | CACGAGCGACGAAACTGTAGATCA | |
| | *AtVAMP723* | Forward | ATGGCTTTCTTGGAACGCGTGAAG | AT2G33110 |
| | | Reverse | GACTCTGAACTACCCAGCATCTCACAT | |
| | *AtCPK1* | Forward | TGTGTTGGACCAAGCAGAAATGGC | AT5G04870 |

| | | | |
|---|---|---|---|
| | Reverse | AAGCCCTGCACTTGACACTCTCTT | |
| *AtCPK2* | Forward | TCGGAGCTGAGCAAGCTTCTTCTT | AT3G10660 |
| | Reverse | AAACGTCGTCCCAAATTGCCCTTG | |
| *AtTPK4* | Forward | AACGAGTCCTCACCGGAAGAAACT | AT1G02510 |
| | Reverse | TCTTGGTCGTTGAAGTTGACGGGA | |
| *AtTPK5* | Forward | TCGTCTTGTTCGGATTCGGGTTCT | AT4G01840 |
| | Reverse | CGATCAATTCTCGCTTCCGCCAAA | |
| *AtVTI11* | Forward | ATACTGTGAGCTCTCGGCGAGTCTTT | AT5G39510 |
| | Reverse | TCTAGCAACTCATCACGAGCAGCA | |
| *AtVTI13* | Forward | AATGGACCTCGAGGCAAGAAACCT | AT3G29100 |
| | Reverse | TCTGTGGTTCGGCCTAAATGGTCT | |
| *AtVTI14* | Forward | AGAAGTTCTTGAAGCTGAAAAGGCGGA | AT5G39630 |
| | Reverse | CACGATTGACCGGCTCTCTTTGACAG | |
| *AtACT2* | Forward | AAGCTGTTCTCTCCTTGTACGCCA | AT3G18780 |
| | Reverse | TCTTCATGCTGCTTGGTGCAAGTG | |
| *AtUBQ10* | Forward | TCACCGGAAAGACAATCACC | BP860797 |
| | Reverse | ACGTACGGCCATCCTCTAG | |

| Gene_ID | Direction | Primer (5'->3') | Submitted GenBank_ID |
|---|---|---|---|
| *CpCPK* | Forward | GAGAGACTTGGTTCTTGCTCTT | KC692920 |
| | Reverse | TCGCCTGATGTTCAGGTTTAG | |

Table S2. Gene-specific primers for the genic annotation in *Carica papaya*.
